# CARM1-mediated OGT arginine methylation promotes non-small cell lung cancer glycolysis by stabilizing OGT

**DOI:** 10.1038/s41419-024-07313-1

**Published:** 2024-12-23

**Authors:** Luyao Lin, Qingxia Yuan, Jiayi Gu, Guangyu Bai, Xianling Cong, Qianying Hu, Jingyao Hou, Xin Jin, Xiangxiang Liu, Baiqu Huang, Yu Zhang, Jun Lu

**Affiliations:** 1 The Key Laboratory of Molecular Epigenetics of Ministry of Education (MOE), Northeast Normal University, 130024 Changchun, China; 2 The Institute of Genetics and Cytology, Northeast Normal University, 130024 Changchun, China; 3 Department of Biobank, China-Japan Union Hospital of Jilin University, 130033 Changchun, China

**Keywords:** Cancer, Molecular biology

## Abstract

O-GlcNAcylation catalyzed by O-GlcNAc transferase (OGT) plays an important role in the regulation of tumor glycolysis. However, the mechanism underlying OGT regulation remains largely unknown. Here, we showed that coactivator associated arginine methyltransferase 1 (CARM1) sensed changes of extracellular glucose levels in non-small cell lung cancer (NSCLC) cells. Increased glucose upregulated CARM1 and OGT. CARM1 methylated OGT at arginine 348, promoting its stability through binding of the deubiquitinase USP9X. The arginine methylation of OGT increased global O-GlcNAcylation levels, thereby promoting glycolysis in NSCLC cells. OGT arginine methylation also upregulated c-Myc expression and promoted the proliferation of NSCLC cells in vitro and in vivo. Consistently, OGT expression was positively correlated with CARM1 in human NSCLC samples. The present findings shed light on the mechanism underlying the stabilization of OGT by arginine methylation in response to changes of glucose concentration. The study also clarified the role of the CARM1-USP9X-OGT axis in glycolysis in NSCLC, providing a potential new target or therapeutic strategy in NSCLC.

## Introduction

Cancer cells reprogram glucose metabolism to aerobic glycolysis rather than oxidative phosphorylation even in the presence of oxygen [1]. Non-small cell lung cancer (NSCLC) is the most common type of lung malignancy, accounting for 85% of all lung cancers [2]. Despite advances in treatment strategies, the five-year survival rate of NSCLC remains low [3]. Tumors from NSCLC patients show increased glycolysis [4], and blocking glycolysis inhibits the proliferation of NSCLC cells [5]. Glycolysis is used as marker for the clinical diagnosis of NSCLC [6]. Therefore, elucidating the mechanisms underlying the regulation of glycolysis in NSCLC is important for clinical practice.

O-GlcNAcylation and OGT are upregulated in most cancers [7]. O-GlcNAcylation catalyzed by O-GlcNAc transferase (OGT) plays an important role in the regulation of tumor glycolysis. c-Myc is a transcription factor, which participates in many cellular processes including cell proliferation and glycolysis. c-Myc could be O-GlcNAcylated by OGT and the O-GlcNAcylation of c-Myc could regulate its stability and function [8, 9]. OGT modifies glycolytic enzymes, transcription factors, and components of the PI3K/Akt/mTOR pathway to promote glycolysis [10, 11]. However, the mechanism underlying the regulation of OGT and its effects in response to changes of extracellular glucose remain unclear.

OGT is regulated by several post-translational modifications (PTMs). The phosphorylation of OGT at Ser3/4 by glycogen synthase kinase 3β [12], at Ser20 by calcium/calmodulin-dependent kinase II [13], and at Tyr976 by insulin receptor kinase activity [14] enhance the activity of OGT. OGT Ser20 phosphorylation by checkpoint kinase 1 leads to its stabilization, which is required for cytokinesis [15]. O-GlcNAcylation of Ser389 in OGT affects its nuclear translocation in HeLa cells [16]. Moreover, the expression of OGT can be regulated by deubiquitinases [17, 18] and ubiquitin ligases [19, 20]. Further research on the PTM of OGT is needed.

Arginine methylation is an important PTM mediated by arginine methyltransferases (PRMTs). PRMTs are involved in glucose metabolism [21]. CARM1 methylates GAPDH to suppress glucose metabolism in liver cancer [21]. In contrast, PKM2 arginine methylation by CARM1 activates glycolysis to promote tumorigenesis in breast cancer [22]. Although the role of CARM1 in different cancers is not clear, studies have confirmed that CARM1 was oncogenic in NSCLC [23]. However, the function of CARM1 in NSCLC glycolysis remains poorly understood.

This study examined the mechanisms underlying the regulation of OGT in response to changes of extracellular glucose. We found that CARM1 sensed the changes of glucose concentration and methylated OGT at arginine 348 (R348). CARM1 stabilized OGT by promoting the binding of USP9X. The arginine methylation of OGT promoted glycolysis and cell proliferation in NSCLC. We also demonstrated a significant correlation between OGT and CARM1 in NSCLC samples. These findings suggested that the CARM1-OGT-USP9X axis could serve as a potential new target for the treatment of NSCLC.

## Materials and methods

### Cell culture

All cell lines (HEK-293T, H1299, and A549) were purchased from the American Type Culture Collection (ATCC, Manassas, VA, USA) and cultured in DMEM (Gibco, Grand Island, NY, USA) containing 10% FBS and antibiotics (100 μg/mL streptomycin and ampicillin) at 37 °C under 5% CO_2_.

### Antibodies, reagents, and plasmids

Complete informations are provided in Supplementary Materials and Methods.

### Reverse transcription, PCR, and real-time PCR

Reverse transcription, PCR, and real-time PCR were performed as described previously [24]. Total RNA was extracted using the RNAiso Plus kit (TaKaRa, Shiga, Japan) following the manufacturer’s instructions. The cDNA was generated using M-MLV Reverse Transcriptase (Promega, Madison, WI, USA) as indicated by the manufacturer.

PCR primer pairs are listed in Supplementary Materials and Methods.

### Western blotting

Western blotting was performed as described previously [25].

Cells were collected and lysed in 1× Laemmli sample buffer. Protein lysates were subjected to SDS-PAGE, transferred to 0.45-μm pore PVDF membranes (Millipore, Burlington, MA, USA), and detected with the ECL reagent (GE Healthcare, Buckinghamshire, UK).

### Co-immunoprecipitation

Co-immunoprecipitation was performed as described previously [24].

Cells were harvested and lysed in buffer A. Lysates were placed in a microcentrifuge at 12,000 × *g* for 10 min at 4 °C; the supernatant was collected and incubated with 3–5 μg of the indicated primary antibodies overnight at 4 °C, followed by addition of 20–40 μL of A/G Magnetic Beads (MCE, Monmouth Junction, NJ, USA) for 2 h. The beads were washed and incubated at 100 °C for 8 min prior to western blot analysis.

### GST pull-down assay

GST pull-down assay was performed as described previously [24].

Flag-PRMT plasmid was transfected into HEK-293T cells for 48 h, and the cell lysates were then incubated with purified GST-OGT fusion proteins plus GST Magnetic Beads (Biolinkedin, China) overnight. Beads were washed with PBS three times prior to western blot analysis.

### Measurement of extracellular acidification rate (ECAR) and lactate

The detailed procedure is described in Supplementary Materials and Methods.

### CCK8 and clone formation assays

The CCK8 assay and colony formation assay were shown in Supplementary Materials and Methods.

### In vitro methylation assay and mass spectrometry analysis

The purified enzyme (GST-CARM1) and substrates (GST-OGT WT; GST-R348K) were incubated in the presence of S-adenosyl-methionine (SAM) for 1 h at 30 °C and then analyzed by western blotting.

Flag-OGT protein was purified from HEK-293T cells followed by SDS–PAGE. Gels were stained with Coomassie Brilliant Blue. The Flag-OGT band was excised and analyzed by liquid chromatography-tandem mass spectrometry (LC-MS/MS) in APTBIO (Shanghai, China).

### Human NSCLC specimens and immunohistochemistry

Human NSCLC samples were provided by China-Japan Union Hospital of Jilin University. The tissue sections were analyzed by immunohistochemistry as previously described [26]. The patient studies were conducted according to the Declaration of Helsinki, and the use of these specimens and data for research purposes was approved by the Ethics Committee of the Hospital.

### Mouse xenograft model

The animal experiments were approved by the Animal Care Committee of Northeast Normal University, China. The stable cell lines H1299-shOGT 3’UTR-WT, H1299-shOGT 3’UTR-R348K, and H1299-shOGT 3’UTR-R348F (3×10^6^ cells suspended in 150 μL PBS) were subcutaneously injected into 5 week-old male BALB/c nude mice (HFK Bioscience, Beijing). After 3 weeks, the mice were sacrificed by euthanasia, and the size and weight of tumors was measured.

### Statistical analysis

Unless specified, data are presented as the mean ± SD. The Student’s *t*-test (two-tailed) was used to determine the statistical significance of differences between groups. *P* < 0.05 was considered statistically significant. Statistical analysis was performed using GraphPad Prism 5 Software (GraphPad Software, La Jolla, CA, USA).

## Results

### OGT is arginine methylated by CARM1 in NSCLC

**Fig. 1:**
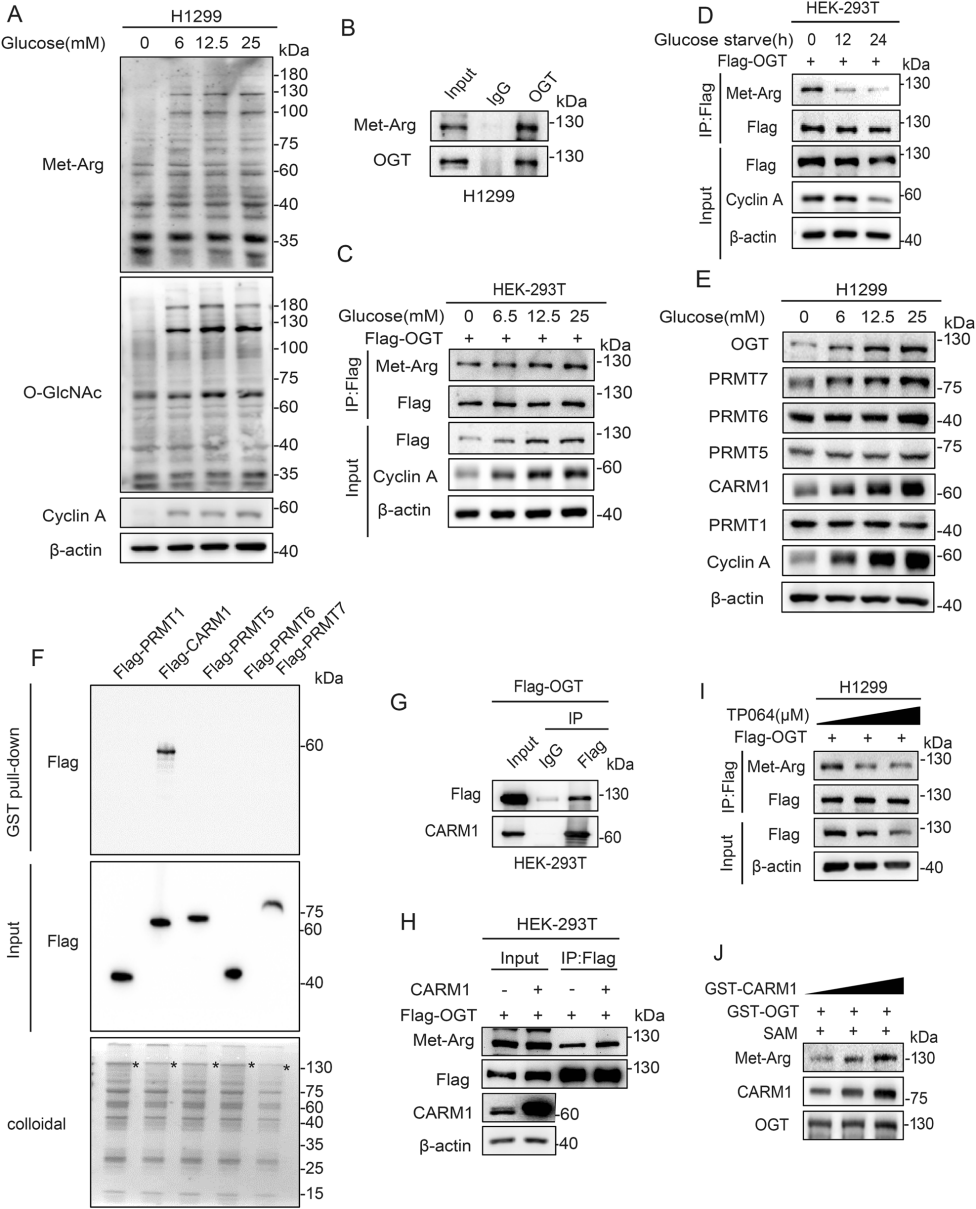
OGT is arginine methylated and the modification is glucose dependent. **A** H1299 cells were cultured for 18 h with different concentrations of glucose as indicated. O-GlcNAcylation and arginine methylation levels were analyzed by western blotting. **B** Endogenous arginine methylation of OGT after Co-IP assay with anti-OGT antibody was detected by western blotting. **C** HEK-293T cells overexpressing Flag-tagged OGT were treated with different concentrations of glucose for 18 h. Co-IP was then performed using anti-Flag antibody. Arginine methylation of immunopurified OGT was detected with antibodies as indicated. **D** HEK-293T cells overexpressing Flag-tagged OGT were treated with glucose starvation for the indicated times. Co-IP was then performed using anti-Flag antibody. Arginine methylation of immunopurified OGT was detected with antibodies as indicated. **E** H1299 cells were cultured with different concentrations of glucose for 18 h. The levels of PRMTs and OGT were analyzed by western blotting. **F** Purified GST-tagged OGT was pulled down with the Flag-PRMTs purified from HEK-293T cells. The amounts of GST-tagged OGT were visualized by Coomassie Blue staining. **G** The interaction between OGT and CARM1 was detected by Co-IP with anti-Flag antibody in HEK-293T cells transfected with Flag-OGT. **H** CARM1 and vector were transformed into HEK-293T cells re-expressing Flag-OGT. Co-IP was then performed using anti-Flag antibody. Arginine methylation of immunopurified OGT was detected with antibodies as indicated. **I** H1299 cells overexpressing Flag-OGT were treated with increasing concentrations of the CARM1 inhibitor TP064 for 24 h as indicated. Cell lysates were immunoprecipitated by anti-Flag antibody and the arginine methylation of OGT was analyzed with antibodies as indicated. **J** Increasing amounts of GST-CARM1 were incubated with GST-OGT in the presence of SAM at 30 °C for 1 h, and arginine methylation of OGT was analyzed by western blotting.

OGT catalyzes the addition of O-GlcNAc to target proteins [27]. To explore whether arginine methylation is involved in the regulation of OGT in response to glucose concentration changes, we first detected global O-GlcNAcylation and arginine methylation associated with changes of extracellular glucose. The results showed that O-GlcNAcylation and arginine methylation levels changed consistently in correlation with changes of glucose (Fig. 1A, S1A-B). Next, we examined whether OGT is regulated by arginine methylation. Arginine modification of OGT decreased after treatment with the PRMT inhibitor Adenosine Dialdehyde (ADOX) in a dose-dependent manner (Fig. S3A). Endogenous OGT was also arginine methylated in H1299 cells (Fig. 1B). OGT arginine methylation increased in correlation with increasing concentrations of glucose (Fig. 1C) and decreased in response to glucose depletion in a time-dependent manner (Fig. 1D). These results indicated that OGT was modified by arginine methylation, and that this modification was glucose dependent.

We then aimed to identify the specific PRMT that arginine methylated OGT and found that only CARM1 interacted strongly with OGT and its expression changed consistently in correlation with the changes of glucose (Fig. 1E-G, S2A, B). The arginine methylation of OGT increased in correlation with the upregulation of CARM1 expression (Fig. 1H). Treatment with the CARM1 inhibitor TP064 decreased OGT arginine methylation in a dose-dependent manner (Fig. 1I), and exposure to exogenous CARM1 increased OGT arginine methylation in a dose-dependent manner in vitro (Fig. 1J). These results demonstrate that CARM1 catalyzed the arginine methylation of OGT, suggesting that CARM1 sensed the changes of glucose and arginine methylated OGT in NSCLC.

### CARM1 methylates OGT at R348

**Fig. 2:**
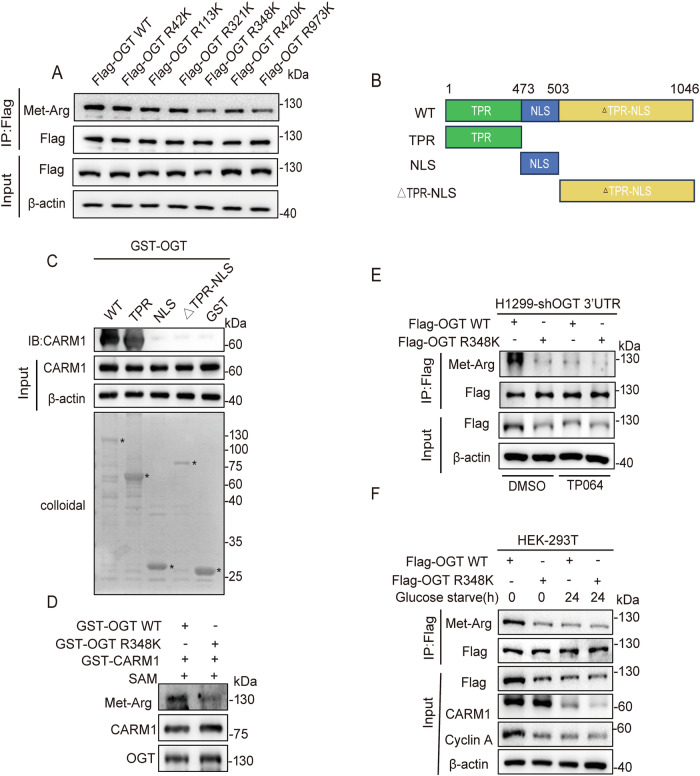
The arginine methylation of OGT at R348 is mediated by CARM1. **A** Six arginine residues were mutated to lysine and transferred into HEK-293T cells. Co-IP was then performed using anti-Flag antibody. Arginine methylation of immunopurified OGT was detected with the indicated antibodies. **B** Schematic diagram of OGT. OGT encodes a polypeptide of 1046 aa with a TPR domain, a NLS domain, and a △TPR-NLS domain. **C** The GST-tagged OGT deletion mutants were subjected to GST pull-down assays in HEK-293T cells. The amounts of GST or GST-tagged mutants were visualized by Coomassie Blue staining (asterisk: GST or GST-OGT mutants). **D** GST-CARM1 was incubated with GST-OGT WT and GST-OGT R348K in the presence of SAM at 30 °C for 1 h. Arginine methylation of OGT was analyzed by western blotting. **E** H1299 cells overexpressing Flag-tagged OGT WT/R348K after knockdown of endogenous OGT were treated with TP064 for 24 h. Cell lysates were immunoprecipitated with anti-Flag antibody, and the arginine methylation of OGT was analyzed with the indicated antibodies. **F** HEK-293T cells overexpressing Flag-tagged OGT WT/R348K were subjected to glucose starvation for 24 h. Cell lysates were immunoprecipitated with anti-Flag antibody, and the arginine methylation of OGT was analyzed with the indicated antibodies.

To define the OGT arginine methylation site(s), we performed liquid chromatography-tandem mass spectrometry (LC-MS/MS) analysis of OGT isolated from HEK-293T-Flag-OGT cells. Six methylated arginine residues (Arg 42/113/321/348/420/973) were identified (Dataset S1A, B, Fig. S4A). To determine the major arginine methylation site(s), the six arginine residues were substituted with lysine and transferred into HEK-293T cells. The result showed that OGT R348K and OGT R973K decreased arginine methylation of OGT (Fig. 2A).

To identify the specific target site of CARM1-mediated OGT arginine methylation, we generated GST-tagged deletion mutants of OGT (Fig. 2B). The GST pull-down assay showed that the mutant TPR domain of OGT showed a strong interaction with CARM1 (Fig. 2C). Structural analysis of OGT indicated that OGT R348 and OGT R973 were located at a considerable distance from each other (Fig. S5A). The R348 site was located in the TPR domain, whereas the R973 site was not, suggesting that CARM1 catalyzed the arginine methylation of OGT at the R348 site. To confirm this, we performed an in vitro methylation assay, and the results showed that the degree of arginine methylation was significantly reduced in OGT R348K compared with OGT WT (Fig. 2D). In addition, treatment with TP064 decreased the arginine methylation of OGT WT, but not that of the OGT R348K mutant (Fig. 2E). Moreover, glucose starvation decreased the arginine methylation of OGT WT but not that of the OGT R348K mutant (Fig. 2F). These results demonstrated that CARM1 arginine methylated OGT at the R348 site.

### Arginine methylation of OGT R348 stabilizes OGT

**Fig. 3:**
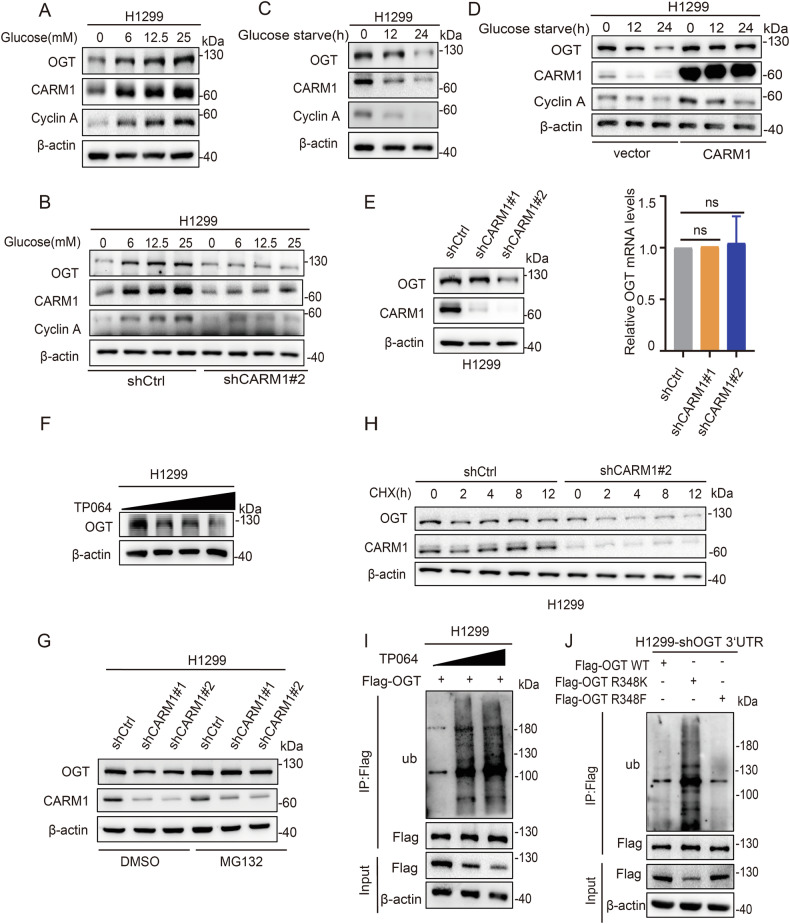
Arginine methylation of OGT at R348 by CARM1 stabilizes OGT. **A** H1299 cells were cultured with different concentrations of glucose for 18 h. CARM1 and OGT levels were analyzed by western blotting. **B** H1299 cells were transfected with CARM1 shRNA#2 or control vector and cultured with different concentrations of glucose for 18 h. OGT and CARM1 levels were analyzed by western blotting. **C** H1299 cells were cultured with glucose starvation for the indicated times. OGT and CARM1 levels were analyzed by western blotting. **D** H1299 cells were transfected with vector or CARM1 and cultured for the indicated times with glucose starvation. CARM1 and OGT levels were analyzed by western blotting. **E** The protein and relative mRNA expression levels of OGT in H1299 cells transfected with CARM1 shRNAs or control were assessed by western blotting and qPCR (error bars represent the mean ± SD, *n* = 3 experimental replicates, ns = not significant, Student’s *t* test). **F**. H1299 cells were treated with the indicated concentrations of TP064 for 24 h. The protein expression levels of OGT in cells were assessed by western blotting. **G** H1299 cells were transfected with control or CARM1 shRNAs and then treated with MG132 or DMSO for 8 h. Cell lysates were analyzed by western blotting. **H** H1299 cells were transfected with CARM1 shRNA#2 or control, and then treated with CHX (100 μg/mL) for the indicated time; OGT protein level was examined by western blotting. **I** H1299 cells expressing Flag-OGT were treated with increasing amounts of TP064 for 24 h. Co-IP assay was performed using anti-Flag and then subjected to western blotting. **J** H1299 cells were transfected with Flag-OGT WT/R348K/R348F after knocking down endogenous OGT. Co-IP assay was performed using anti-Flag and then subjected to western blotting.

After demonstrating that CARM1 methylated OGT at the R348 site, we examined the roles of arginine methylation. We first showed that OGT and CARM1 were consistently upregulated in correlation with increasing glucose concentration, and knockdown of CARM1 inhibited the upregulation of OGT (Fig. 3A, B, S6A, B). Similarly, OGT and CARM1 were downregulated concomitant with increasing starvation time, and overexpression of CARM1 inhibited OGT downregulation (Fig. 3C, D, S6C, D). Knockdown of CARM1 downregulated OGT protein expression (Fig. 3E, S6E), whereas the mRNA levels hardly changed (Fig. 3E). In addition, TP064 treatment downregulated OGT protein expression (Fig. 3F, S6F). Taken together, these results suggested that CARM1-mediated OGT arginine methylation was responsible for its stability.

OGT is degraded by the proteasome, which led us to hypothesize that CARM1 modulates the stability of OGT through the ubiquitin-proteasome pathway. Treatment with the proteasome inhibitor MG132 reversed the inhibitory effect of CARM1 knockdown on OGT (Fig. 3G, S6G), and CARM1 knockdown decreased the half-life of OGT in the presence of the protein synthesis inhibitor cycloheximide (CHX) (Fig. 3H, S6H). Furthermore, treatment with TP064 increased the ubiquitination of OGT in a dose-dependent manner in H1299 cells (Fig. 3I). These results supported the notion that CARM1 stabilized OGT by decreasing its ubiquitination. We therefore examined whether the methylation of OGT R348K contributed to OGT stability. We found that ubiquitination levels were higher in the OGT R348K mutant than in OGT WT and the OGT R348F (a mimic of constitutive methylated status) mutant (Fig. 3J). These results indicated that CARM1-mediated arginine methylation of OGT R348 promoted OGT stabilization.

### Arginine methylation of OGT R348 promotes OGT stability through USP9X

**Fig. 4:**
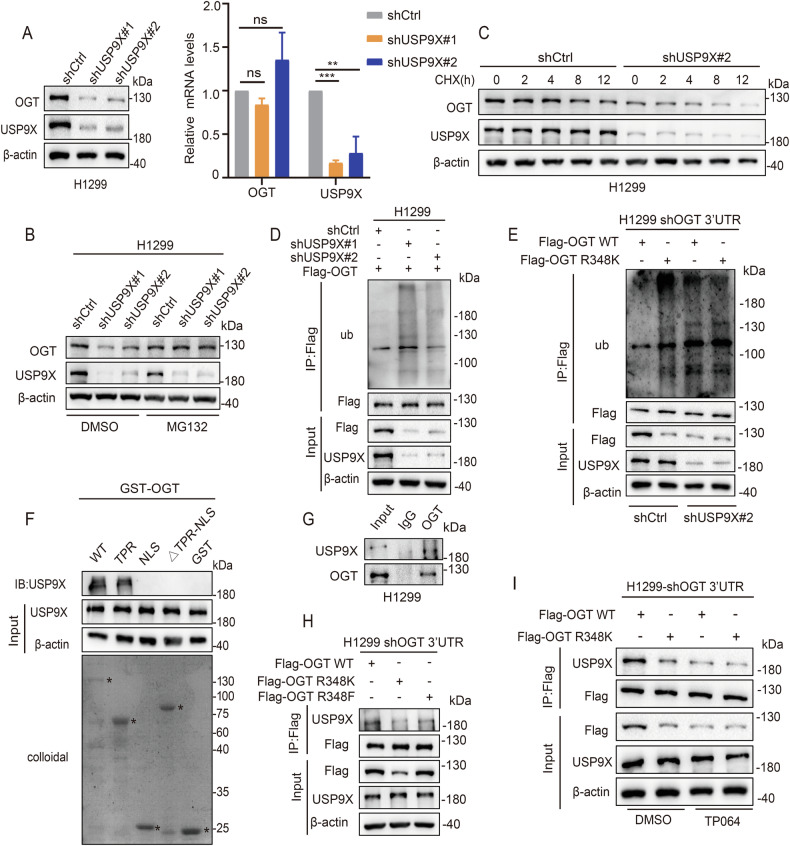
Arginine methylation of OGT at R348 by CARM1 promotes OGT stability through USP9X. **A** The protein and relative mRNA expression levels of OGT in H1299 cells transfected with USP9X shRNAs or control were assessed by western blotting and qPCR (error bars represent the mean ± SD, *n* = 3 experimental replicates, ns = not significant, ^**^P < 0.01, ^***^P < 0.001, Student’s *t* test). **B** H1299 cells were transfected with control or USP9X shRNAs and then treated with MG132 or DMSO for 8 h; cell lysates were analyzed by western blotting. **C** H1299 cells were transfected with USP9X shRNA#2 or control, and then treated with CHX (100 μg/mL) for the indicated times; OGT protein level was examined by western blotting. **D** H1299 cells expressing Flag-OGT were transfected with control or USP9X shRNAs. Co-IP assay was performed using anti-Flag and then subjected to western blotting. **E** H1299 cells expressing Flag-OGT WT and Flag-OGT R348K after knockdown of endogenous OGT were transfected with control or USP9X shRNA#2. Co-IP assay was performed using anti-Flag and then subjected to western blotting. **F** The GST-tagged OGT deletion mutants were subjected to GST pull-down assays with HEK-293T cells. The amounts of GST or GST-tagged mutants were visualized by Coomassie Blue staining (asterisk: GST or GST-OGT mutants). **G** The interaction between OGT and USP9X was detected by Co-IP with anti-OGT antibody in H1299 cells. **H** H1299 cells were transfected with Flag-OGT WT/R348K/R348F after knockdown of endogenous OGT. Co-IP assay was performed using anti-Flag and then subjected to western blotting. **I** H1299 cells were transfected with Flag-OGT WT/R348K after knockdown of endogenous OGT then treated with TP064 for 24 h. Co-IP assay was performed using anti-Flag and then subjected to western blotting.

Deubiquitination plays an important role in protein stabilization. We identified several USPs family members (USP7, USP9X, USP10, USP15) that could bind to OGT by reviewing OGT binding mass spectrograms uploaded from the literature [28, 29] .We found that OGT downregulation was most pronounced after knockdown of USP9X (Fig. S7A). Knocking-down USP9X significantly reduced the protein level of OGT, whereas the level of mRNA was almost unchanged (Fig. 4A). MG132 reversed the inhibitory effect of USP9X knockdown on OGT (Fig. 4B). In addition, USP9X knockdown shortened the half-life of OGT (Fig. 4C), and OGT ubiquitination increased after USP9X knockdown (Fig. 4D). The OGT R348K mutant showed higher levels of ubiquitination than OGT WT, and knockdown of USP9X increased the ubiquitination of OGT WT, but not that of the OGT R348K mutant (Fig. 4E). These results demonstrated that USP9X was the deubiquitinating enzyme of OGT and stabilized OGT through OGT R348 arginine methylation.

Next, we examined the mechanism by which arginine methylation of OGT affects its stability through USP9X. We first confirmed that USP9X binds to OGT in H1299 cells by endogenous Co-IP assay (Fig. 4G). The results of GST pull-down showed that the mutant TPR domain of OGT interacted strongly with USP9X (Fig. 4F). The interaction between the deubiquitinating enzyme USP9X and OGT was attenuated in the OGT R348K mutant (Fig. 4H). Moreover, TP064 treatment significantly weakened the binding interaction between OGT WT and USP9X, but did not affect that of OGT R348K (Fig. 4I). These results confirmed that the arginine methylation of OGT R348 by CARM1 stabilized OGT by promoting binding to USP9X.

### The arginine methylation of OGT R348 promotes glycolysis in NSCLC

**Fig. 5:**
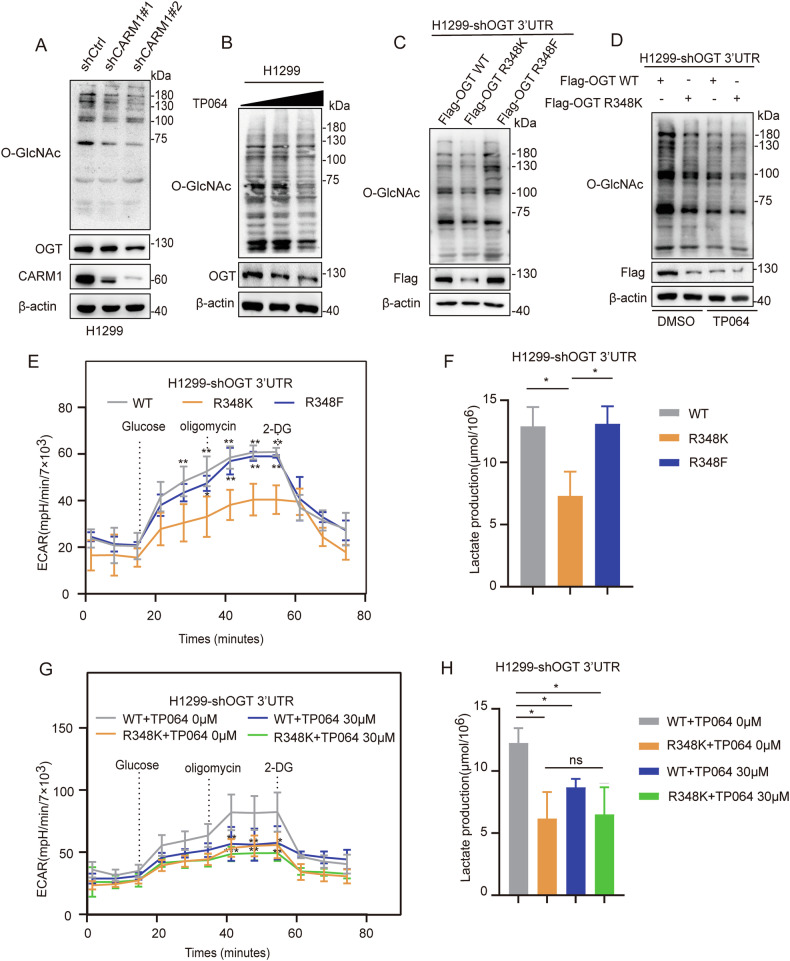
Arginine methylation of OGT at R348 promotes glycolysis in NSCLC. **A** H1299 cells transfected with CARM1 shRNAs or control were assessed by western blotting. O-GlcNAcylation levels in cells were assessed by western blotting. **B** H1299 cells were treated with increasing amounts of TP064 for 24 h. O-GlcNAcylation levels in cells were assessed by western blotting. **C** H1299 cells were transfected with Flag-OGT WT /R348K/R348F after knocking down endogenous OGT. O-GlcNAcylation levels of H1299 cells were assessed by western blotting. **D** The O-GlcNAcylation level was measured in H1299 cells re-expressing OGT WT/R348K after knockdown of endogenous OGT and treatment with TP064 for 24 h. O-GlcNAcylation levels in cells were assessed by western blotting. **E**, **F** ECAR (**E**) and lactic acid production (**F**) were measured in H1299 cells re-expressing OGT WT/R348K/R348F after knockdown of endogenous OGT (error bars indicate the mean ± SD, *n* = 3 experimental replicates, ^*^P < 0.05, ^**^P < 0.01, Student’s *t* test). **G**, **H** ECAR (**G**) and lactic acid production (**H**) were measured in H1299 cells re-expressing OGT WT/R348K after knockdown of endogenous OGT and treatment with TP064 for 24 h (error bars indicate the mean ± SD, *n* = 3 experimental replicates, ns = not significant, ^*^P < 0.05, ^**^P < 0.01, ^***^P < 0.001, Student’s *t* test).

The results showing that arginine methylation of OGT can affect the stability of OGT led us to examine whether it can affect the O-GlcNAcylation modification. We found that inhibition of CARM1 decreased global O-GlcNAcylation levels (Fig. 5A, B). O-GlcNAcylation levels were significantly lower in the OGT R348K mutant than in OGT WT and the R348F mutant (Fig. 5C). TP064 treatment significantly decreased O-GlcNAcylation in OGT WT, but not in the OGT R348K mutant (Fig. 5D).

We also investigated the effect of OGT methylation on glycolysis. The results showed that lactic acid production and ECAR were decreased in the OGT R348K mutant compared with OGT WT and the R348F mutant (Fig. 5E, F). Inhibition of CARM1 by TP064 significantly decreased lactic acid production and ECAR in OGT WT, whereas the OGT R348K mutant did not show significant changes (Fig. 5G, H). These results demonstrated that arginine methylation of OGT R348 by CARM1 promoted glycolysis in NSCLC.

### Arginine methylation of OGT R348 promotes non-small cell lung cancer proliferation

**Fig. 6:**
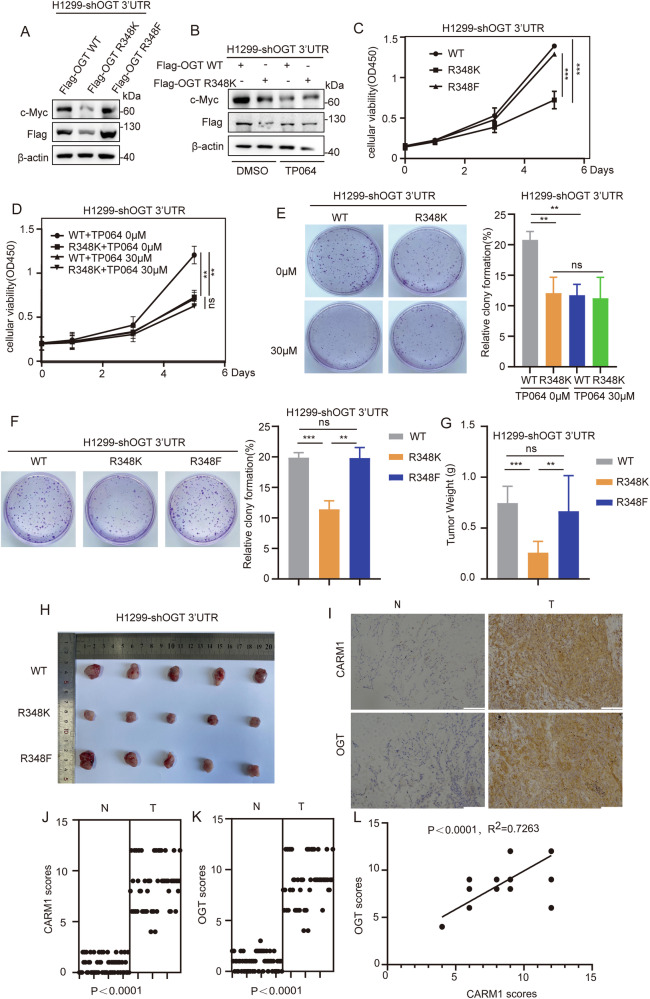
Arginine methylation of OGT at R348 promotes NSCLC proliferation. **A** H1299 cells were transfected with Flag-OGT WT /R348K/R348F after knockdown of endogenous OGT. The level of c-Myc in H1299 cells was assessed by western blotting. **B** The level of c-Myc was measured in H1299 cells re-expressing OGT WT/R348K after knockdown of endogenous OGT and then treated with TP064 for 24 h. c-Myc expression was assessed by western blotting. **C**, **F** H1299 cells were transfected with Flag-OGT WT/R348K/R348F after knockdown of endogenous OGT. Representative images of CCK8 (**C**) and colony formation (**F**) are shown. All data points are included (error bars represent the mean ± SD, *n* = 3 experimental replicates, ^**^P < 0.01, ^***^P < 0.001, ns = not significant, Student’s *t* test). **D**, **E** H1299 cells expressing OGT WT/R348K after knockdown of endogenous OGT were treated with TP064 for 24 h. Representative images of CCK8 (**D**) and colony formation (**E**) are shown. All data points are included (error bars represent the mean ± SD, *n* = 3 experimental replicates, ^**^P < 0.01, ns = not significant, Student’s *t* test). **G**, **H** H1299-shOGT-WT/R348K/R348F cells (3 × 10^6^) were injected into the skin of 5-week-old-male nude mice, and 3 weeks later, mice were sacrificed by euthanasia. Tumor size (**H**) and weight (**G**) were measured (error bars indicate the mean ± SD, *n* = 5 mice for each group, ns=not significant, ^**^P < 0.01, ^***^P < 0.001, Student’s *t* test). **I** Representative images of immunohistochemical staining of OGT and CARM1 in human non-small cell lung cancer samples. Scale bars, 100 μm. **J**,**K** The expression levels of OGT (**K**) and CARM1 (**J**) in human non-small cell lung cancer tissues and adjacent tissues were measured (*n* = 59 non-small cell lung tumor samples, *P* < 0.0001, Student’s *t* test). **L** The correlation of OGT with CARM1 was statistically significant in human non-small cell lung cancer samples (*n* = 59 human non-small cell lung cancer samples; *R*^2^ = 0.7263, *P* < 0.0001; Pearson’s correlation test). The immunohistochemistry score was calculated according to Feng et al. [26].

OGT can enhance the stability of c-Myc, thus promoting cell proliferation [30]. We found that c-Myc expression levels were significantly lower in the OGT R348K mutant than in OGT WT and the R348F mutant (Fig. 6A). The expression of c-Myc in OGT WT was significantly higher than that in the OGT R348K mutant, and treatment with TP064 significantly downregulated c-Myc in OGT WT, whereas the change in OGT R348K was not significant (Fig. 6B).

We then examined the effect of arginine methylation of OGT on the proliferative capacity of NSCLC cells. The results showed that cell proliferation rates were lower in the OGT R348K mutant than in OGT WT and the R348F mutant (Fig. 6C, F). Moreover, inhibition of CARM1 caused a more significant inhibition of proliferation in OGT WT than in the R348K mutant (Fig. 6D, E). To evaluate the roles of OGT arginine methylation of NSCLC in vivo, H1299-shOGT 3’-UTR-WT/R348K/R348F cells were injected subcutaneously into nude mice. The results showed that tumor size and weight were lower in mice injected with OGT R348K mutant cells than in those injected with OGT WT and the R348F mutant (Fig. 6G, H). These results confirmed that the arginine methylation modification of OGT promoted the proliferation of NSCLC.

To investigate the clinical relevance of OGT and CARM1, immunohistochemical staining was performed in human NSCLC samples. CARM1 and OGT expression was higher in cancer tissues than in paracancer tissues (Fig. 6I–K). Data analysis showed that the correlation between OGT and CARM1 was statistically significant (Fig. 6L). CARM1 expression was positively correlated with OGT expression in NSCLC.

## Discussion

OGT is the only glycosyltransferase catalyzing O-GlcNAcylation, and thus plays an important role in the regulation of tumor cell metabolism and cell proliferation [31]. OGT has been shown to promote tumor development [32], and the results of this study confirmed that OGT is an oncogenic factor in NSCLC. We first described the role of OGT arginine methylation in NSCLC, as well as the molecular events involved in this process. We demonstrated that CARM1 sensed the changes of extracellular glucose and methylated OGT at the R348 site. Methylation at R348 stabilized OGT by promoting binding of the deubiquitinase USP9X, and accelerated the glycolysis and proliferation of NSCLC. This finding revealed a new function of the CARM1-OGT-USP9X axis in NSCLC carcinogenesis.

CARM1 is the substrate of OGT and functions to alter OGT substrate specificity in vitro [33]. Abnormal expression of OGT inhibits the phosphorylation of CARM1, resulting in incorrect localization of CARM1 during mitosis in HeLa cells [34]. In this study, we confirmed that OGT was the new substrate of CARM1. CARM1 methylated OGT and increased its stability in response to changes of extracellular glucose in NSCLC. However, the mutual regulation between OGT and CARM1 during tumor progression needs to be further studied.

In hepatocellular carcinoma, CARM1 acts as a tumor suppressor, sensing changes of glucose in an AMPK-dependent form. It is upregulated under glucose starvation conditions [21, 35]. We found CARM1 was downregulated in response to glucose starvation in NSCLC cells (Fig. 3C). It has been reported that the level of CARM1 increases during glucose deprivation in MEF, HeLa and HepG2 cells. Glucose starvation activates AMPKα2 in the nucleus, leading to transcriptional repression of SKP2 via FOXO3a phosphorylation. Reduction of SKP2 expression in turn leads to increased levels of CARM1. CARM1 is ubiquitinated by the SKP2-containing E3 ligase complex under nutrient-rich condition [36]. However, it has also been reported SKP2 is overexpressed in NSCLC cells and tissues [37] while CARM1 expression is higher in NSCLC [23]. These suggested that the level of CARM1 in NSCLC cells was not regulated by SKP2. The mechanism that CARM1 was down-regulated during glucose starvation in NSCLC cells may also not be due to the axis of AMPK–SKP2–CARM1. This difference may be due to differences in cell types. To investigate the mechanism that CARM1 was down-regulated during glucose starvation in NSCLC cells, we examined the effect of glucose starvation on CARM1 mRNA in H1299 cells. The result showed the CARM1 mRNA had no changes with the time of glucose starvation (Fig. S8B). We also found the CARM1 mRNA had no changes with the up-regulation of glucose concentration (Fig. S8A). These results indicated that the regulation of CARM1 by glucose conditions is carried out at the post-transcriptional level. The specific mechanism needs to be further explored. CARM1 promoted the proliferation of NSCLC cells (Fig. 6D, E), and CARM1 expression was higher in human NSCLC samples than in paracancerous tissues (Fig. 6J). These results suggested that CARM1 was an oncogenic factor in NSCLC. This is consistent with the effect of CARM1 on promoting the progression of NSCLC [23, 38]. These observations suggested that the roles of CARM1 in tumor development were complex and tumor type-dependent.

Glucose depletion induces the phosphorylation of the co-chaperone unconventional prefoldin RPB5 interactor (URI) at Ser-371, releasing protein phosphatase γ to inhibit OGT, thereby promoting hepatoma carcinoma cell survival under metabolic stress [39]. Here, we confirmed that the CARM1 and OGT proteins were upregulated with increasing glucose concentration. Methylated OGT R348 by CARM1 increased OGT stability and accelerated the proliferation of NSCLC. The OGT R973K mutant also showed lower arginine methylation than OGT WT (Fig. 2A). However, CARM1 interacted with OGT at the OGT TPR domain (1–473 aa), which was at a considerable distance from the OGT R973 site (Fig. 2C, S5A). Therefore, we focused on the OGT R348 site rather than on the OGT R973 site. OGT may also be arginine methylated at R973 in a process independent from CARM1. Further studies are needed to determine which PRMT mediates the methylation of OGT R973 and the role of the OGT R973 site.

Although OGT can be ubiquitinated [19, 20, 40, 41], the expression of OGT increased in most tumor cells. There are several mechanisms of OGT stabilization in cells. Deubiquitinating enzymes (DUBs) antagonize ubiquitination to control the stability of substrates [42]. The deubiquitinase EIF3H promotes hepatocellular carcinoma progression by stabilizing OGT [17]. USP8 stabilizes OGT by inhibiting the K48-specific polyubiquitination of OGT at residue K117 in HCC [18]. BAP1 mediates the poly-deubiquitination of OGT to prevent its proteasomal degradation in NCI-H226 mesothelioma cells [43]. In this report, we demonstrated that the arginine methylation of OGT by CARM1 enhanced the stability of OGT through binding of USP9X, which inhibited the polyubiquitination of OGT in NSCLC cells. We thus elucidated a novel OGT deubiquitination mechanism and provided an explanation for the high expression of OGT in NSCLC.

**Fig. 7:**
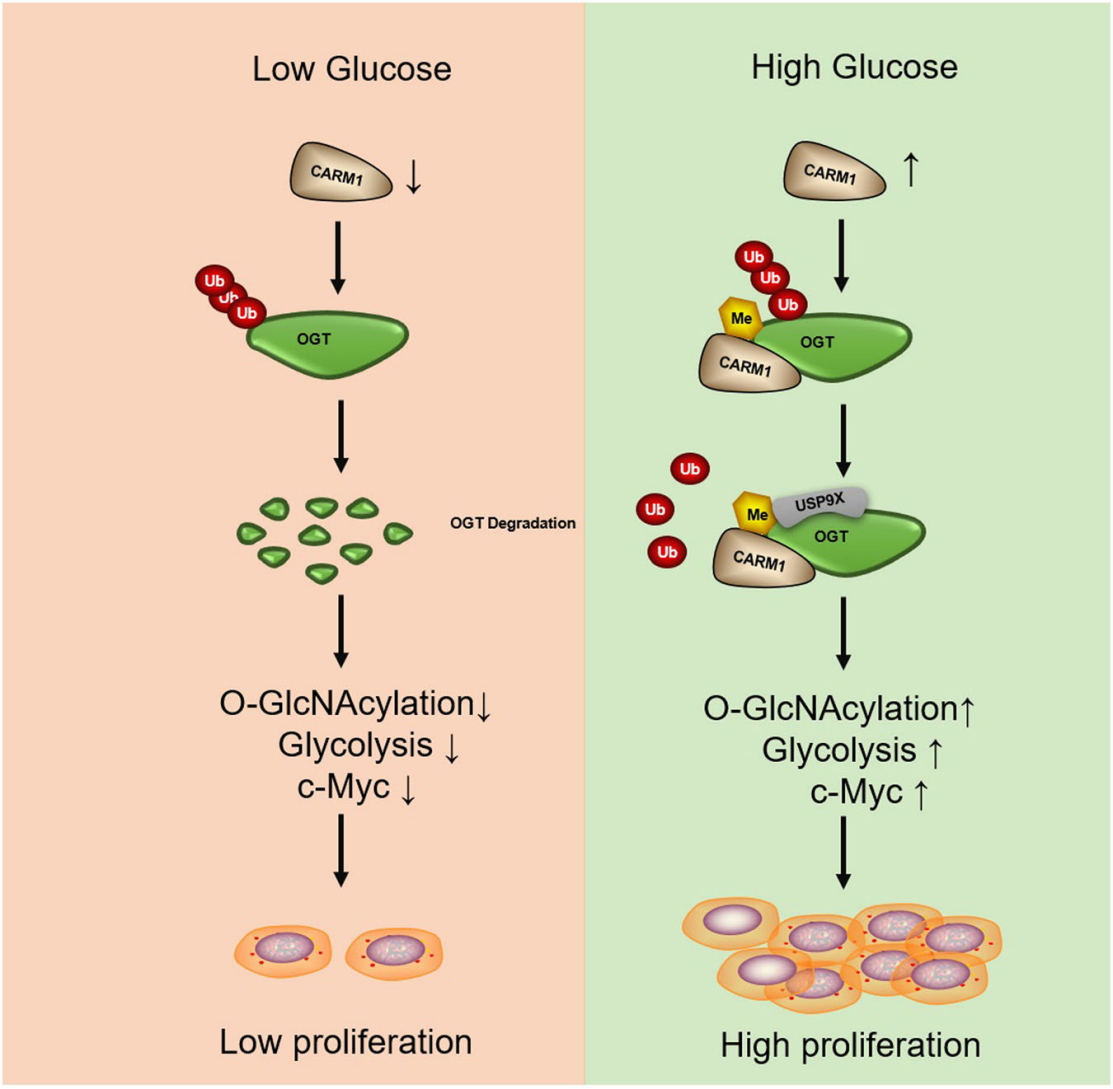
A working model. CARM1 signals the increase of extracellular glucose and arginine methylates OGT at the R348 site, stabilizing OGT by promoting the binding od USP9X. This leads to increased glycolysis and c-Myc expression, which increases the proliferation of NSCLC cells in vitro and in vivo.

In this study, we showed that CARM1 sensed the increase in extracellular glucose and methylated OGT at the R348 site, stabilizing OGT by promoting the binding of USP9X. This led to increased glycolysis and the upregulation of c-Myc, which promoted the proliferation of NSCLC cells in vitro and in vivo (Fig. 7). We identified a novel deubiquitinating enzyme for OGT, and suggested a mechanism underlying the upregulation of OGT in NSCLC. We also elucidated the mechanism underlying the regulation of OGT in response to changes of extracellular glucose. In conclusion, CARM1-mediated OGT methylation was necessary for the progression of NSCLC, and the removal of this modification may provide a new strategy for the treatment of NSCLC.

## Supplementary information

FigS1

FigS2

FigS3

FigS4

FigS5

FigS6

FigS7

FigS8

Supplementary Materials and Methods

Dataset S1A peptides

Dataset S1B proteins

WB blot

## Data Availability

The data supporting the findings of this study are available from the corresponding author upon reasonable request.
