# Supplementary figures and images for: CARM1-mediated OGT arginine methylation promotes non-small cell lung cancer glycolysis by stabilizing OGT
Source: Cell Death Dis. 2024 Dec 23;15(12):927. doi: 10.1038/s41419-024-07313-1 (PMC11666572; doi:10.1038/s41419-024-07313-1)

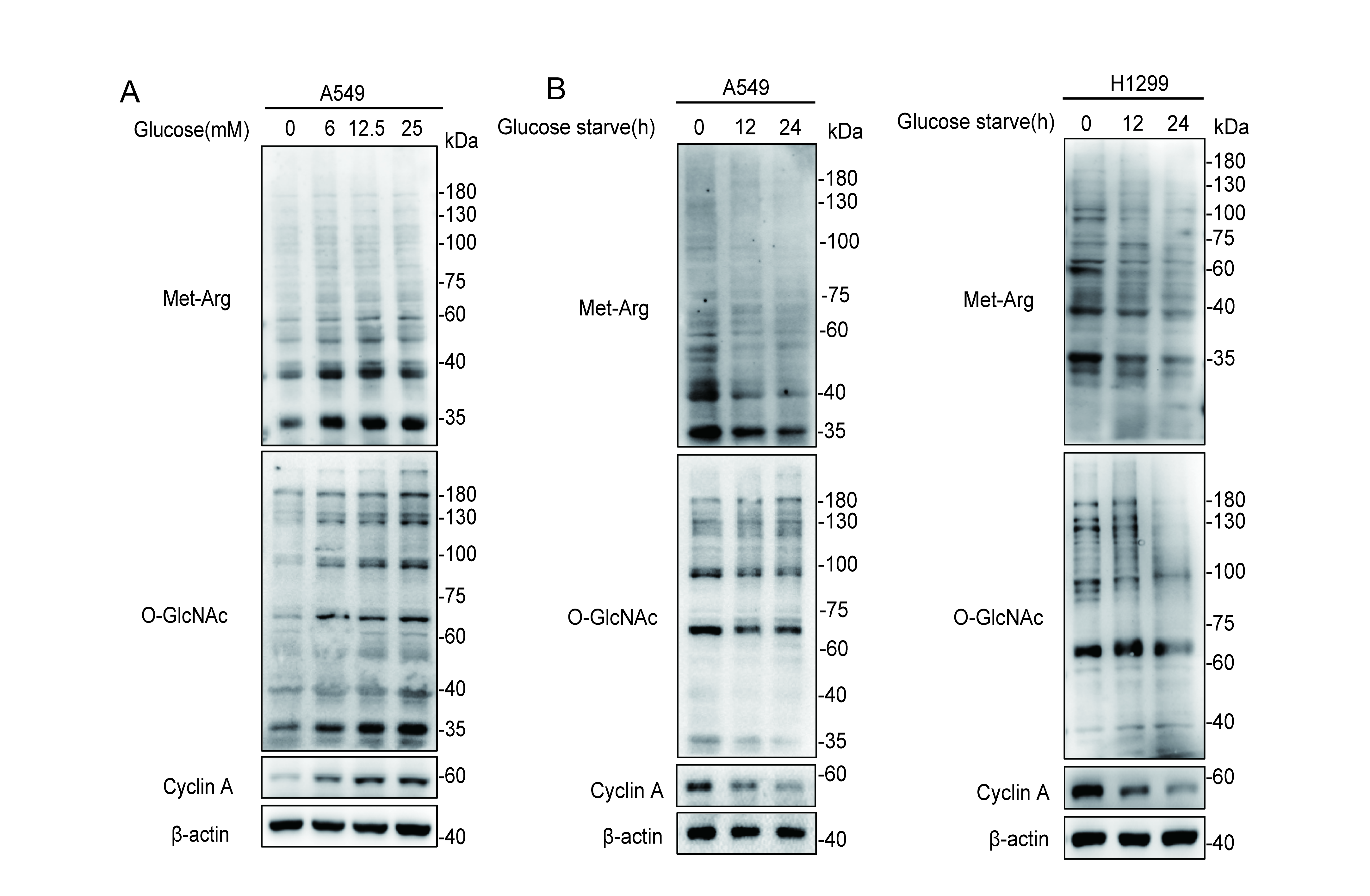

Supplement: Supplementary file 1 — FigS1 [file 41419_2024_7313_MOESM1_ESM.tif]

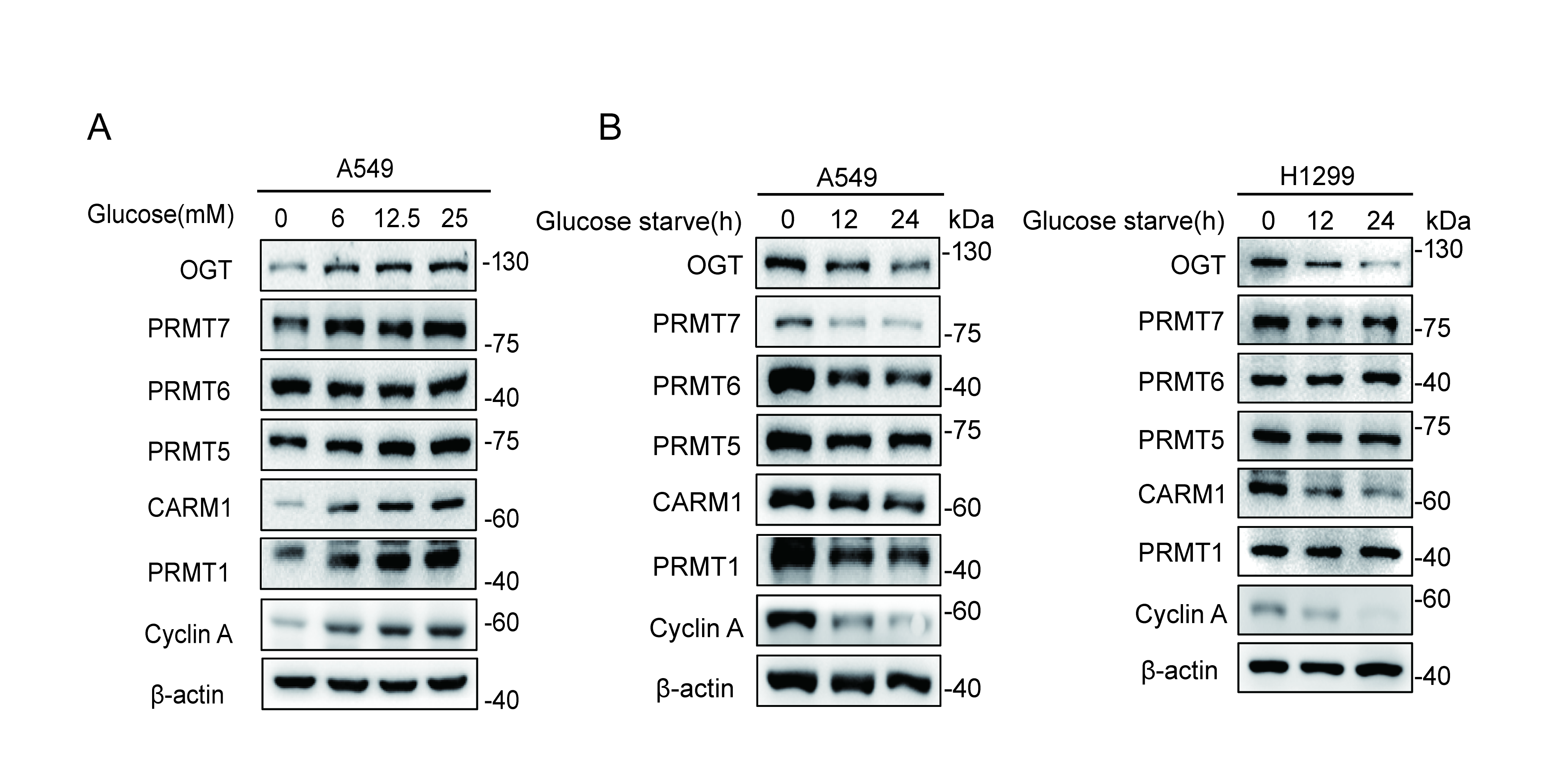

Supplement: Supplementary file 2 — FigS2 [file 41419_2024_7313_MOESM2_ESM.tif]

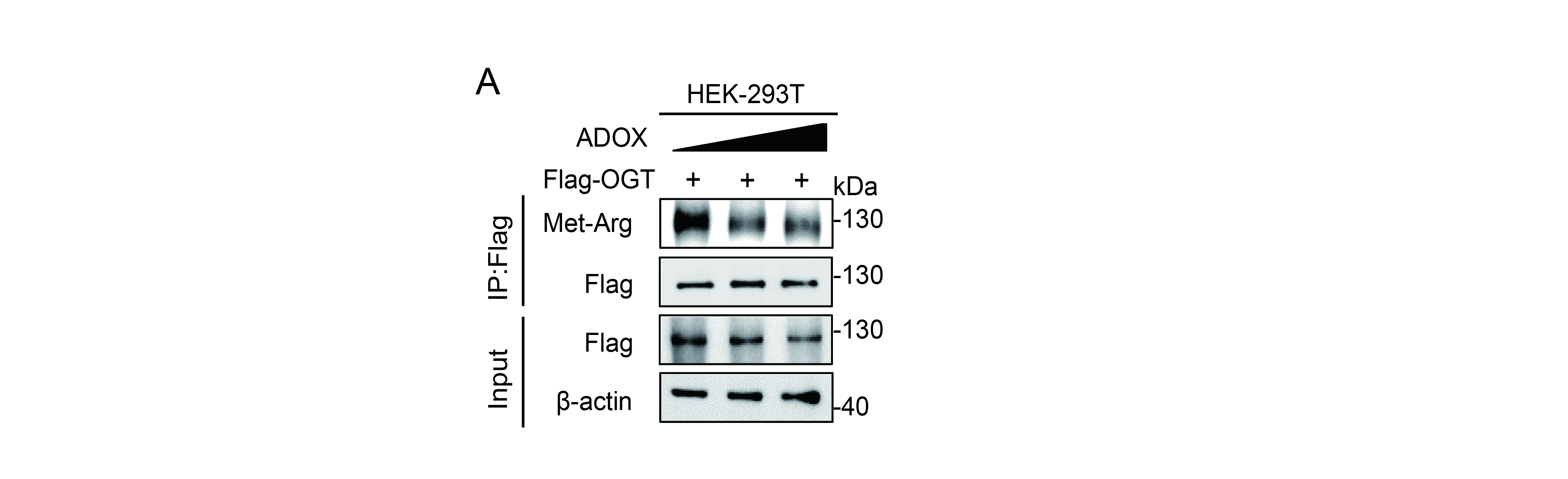

Supplement: Supplementary file 3 — FigS3 [file 41419_2024_7313_MOESM3_ESM.tif]

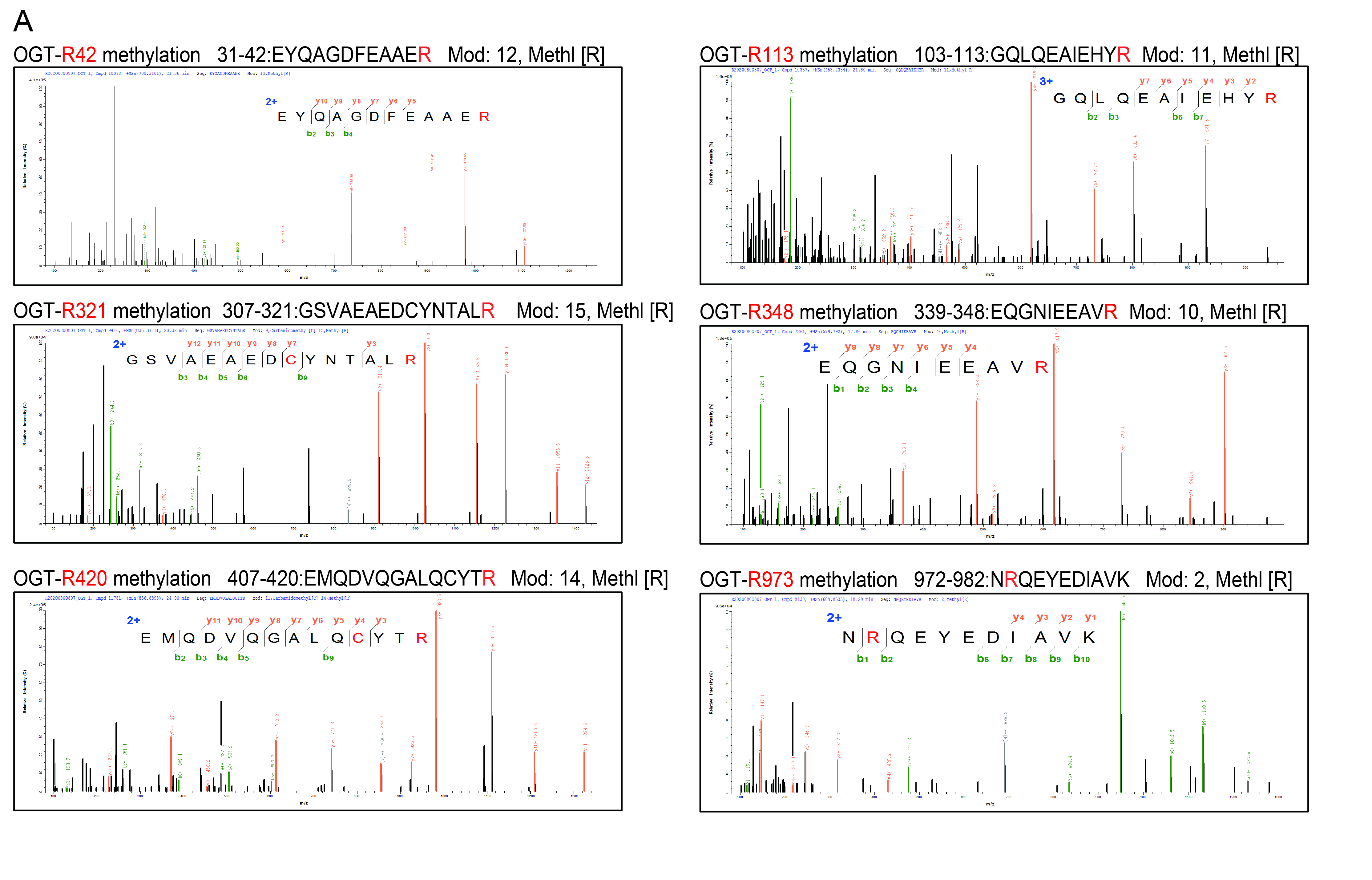

Supplement: Supplementary file 4 — FigS4 [file 41419_2024_7313_MOESM4_ESM.tif]

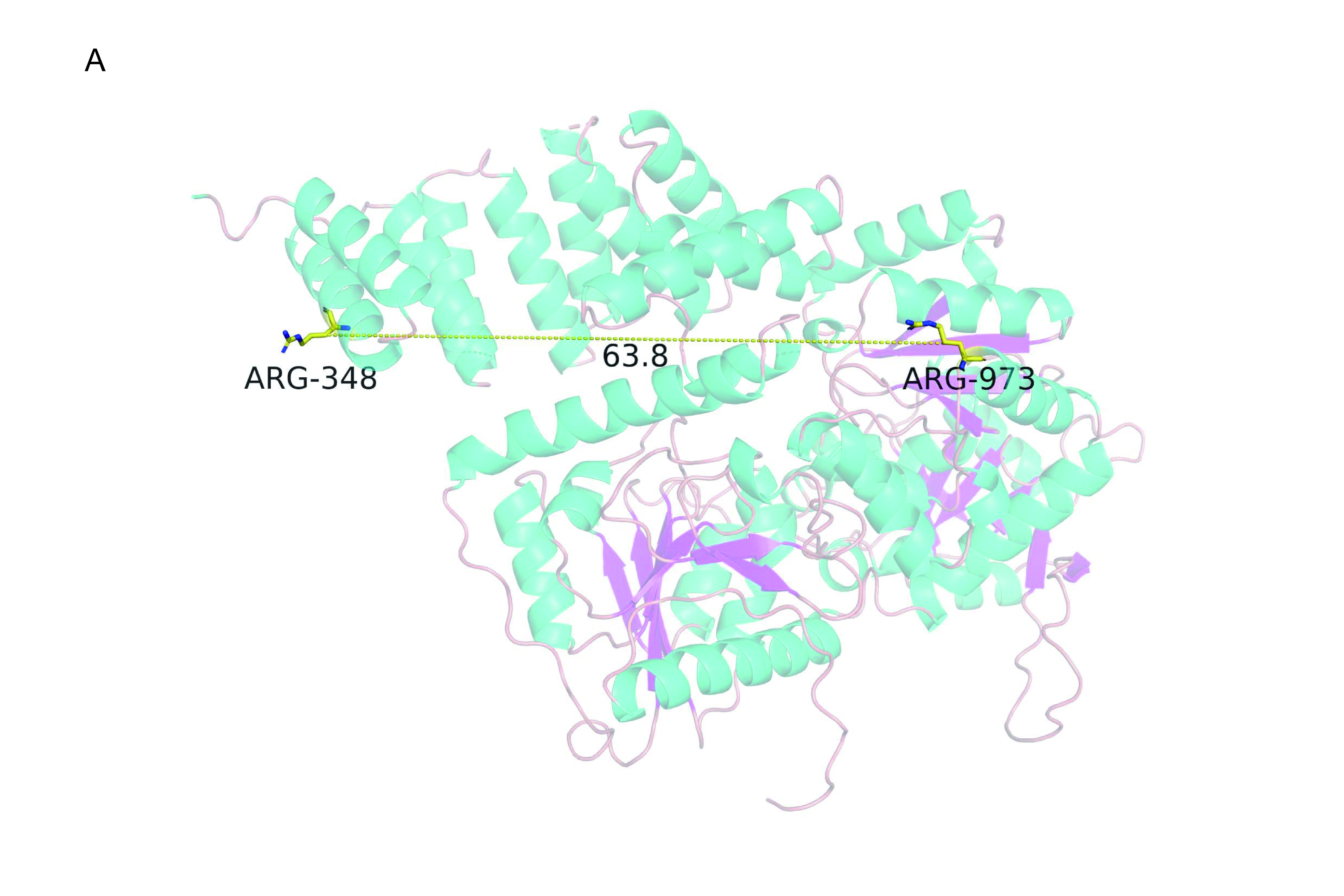

Supplement: Supplementary file 5 — FigS5 [file 41419_2024_7313_MOESM5_ESM.tif]

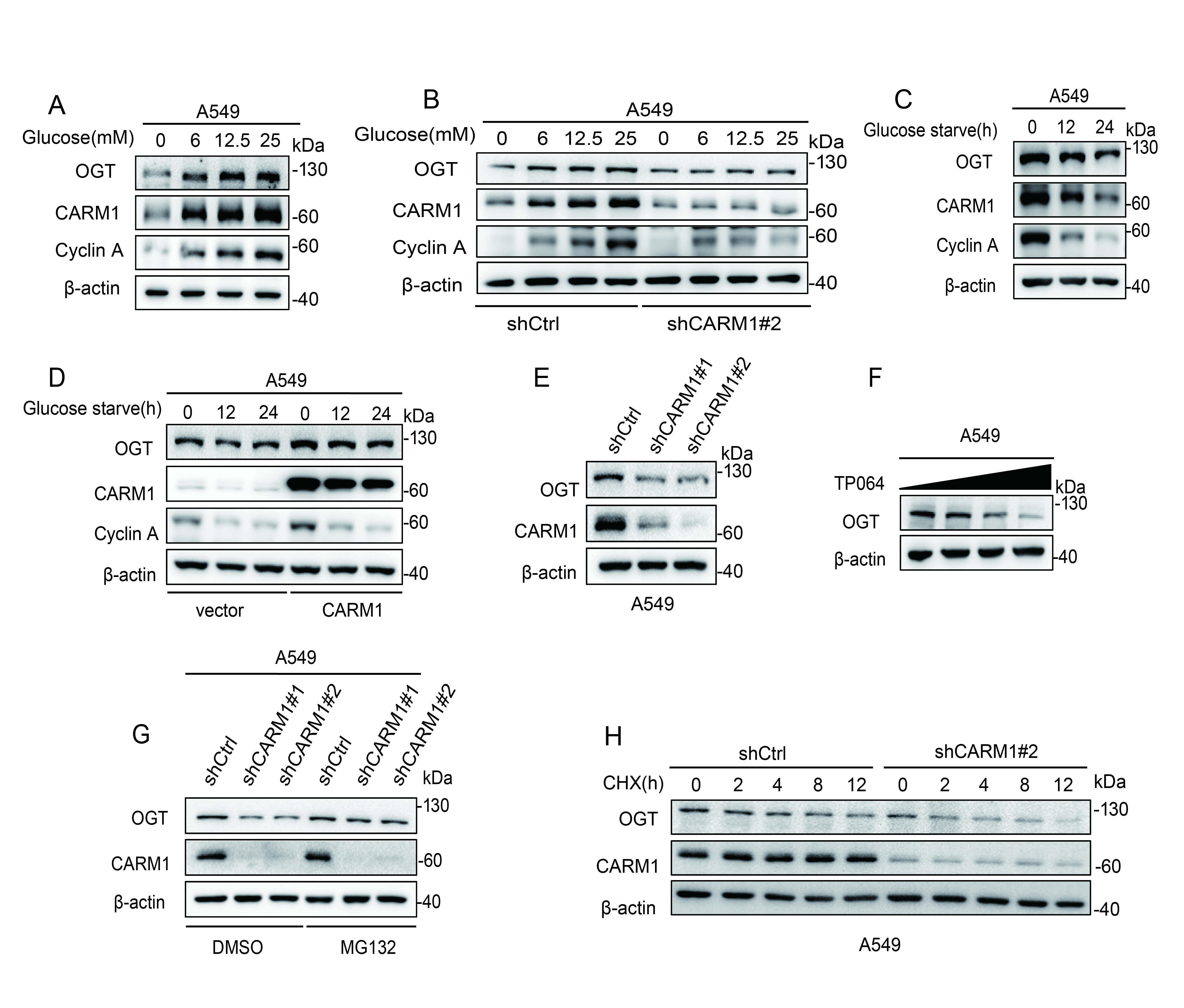

Supplement: Supplementary file 6 — FigS6 [file 41419_2024_7313_MOESM6_ESM.tif]

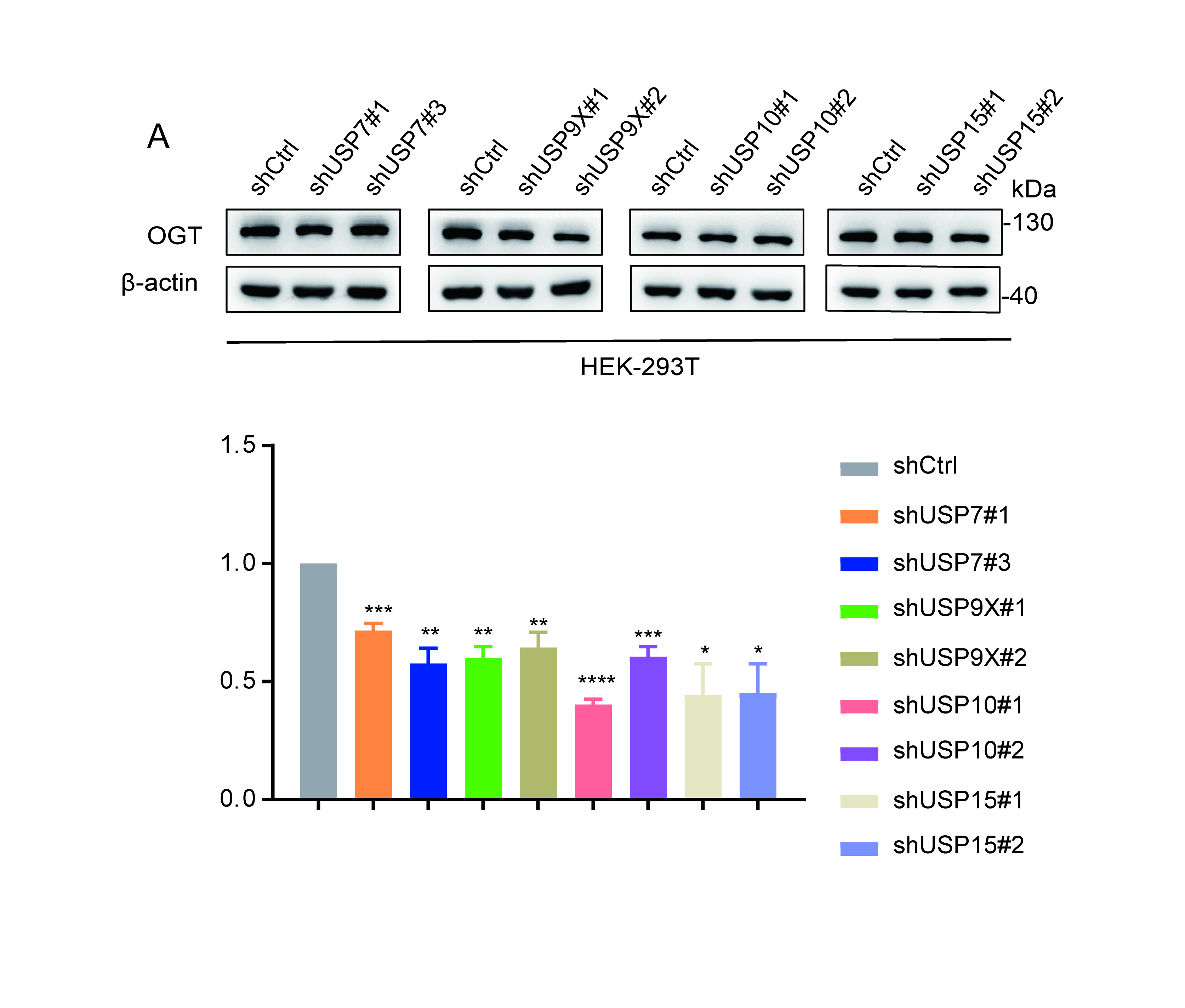

Supplement: Supplementary file 7 — FigS7 [file 41419_2024_7313_MOESM7_ESM.tif]

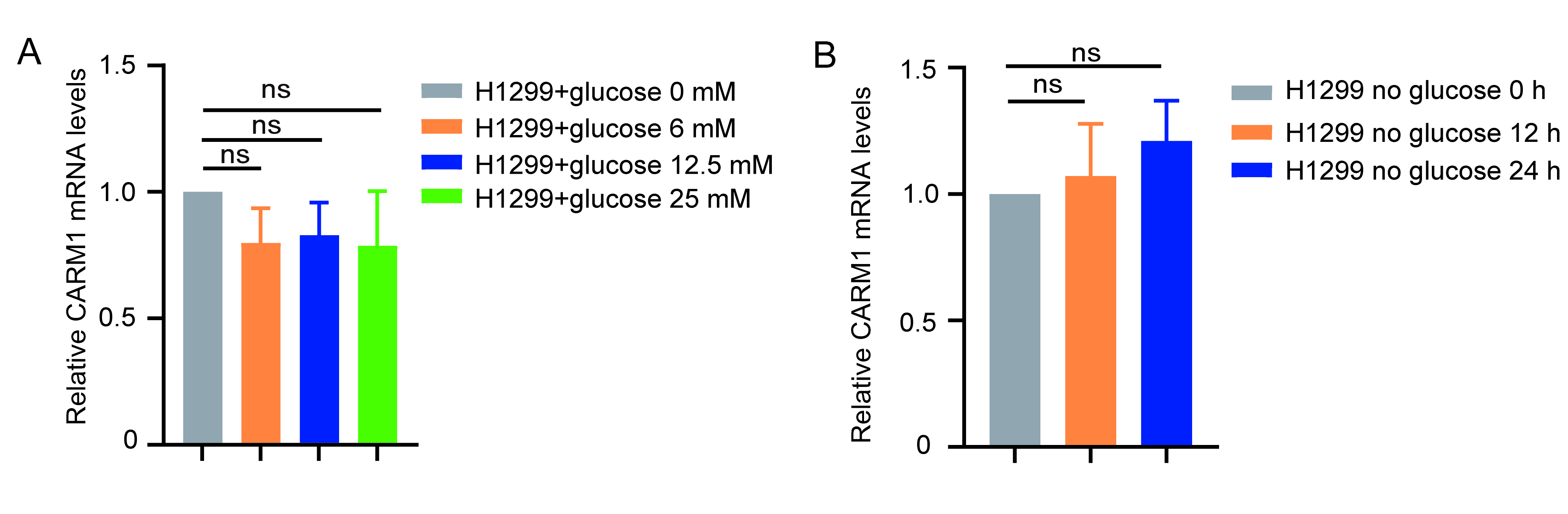

Supplement: Supplementary file 8 — FigS8 [file 41419_2024_7313_MOESM8_ESM.tif]

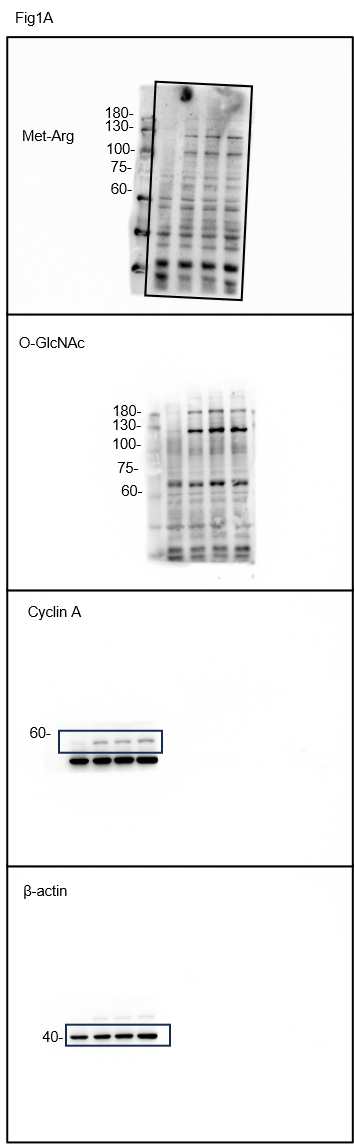

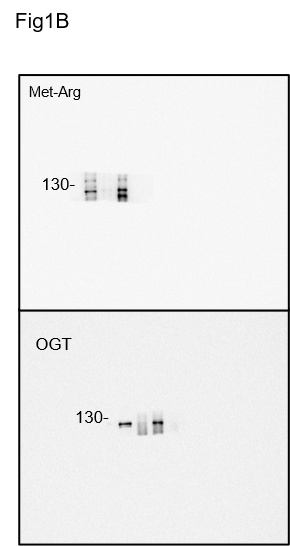

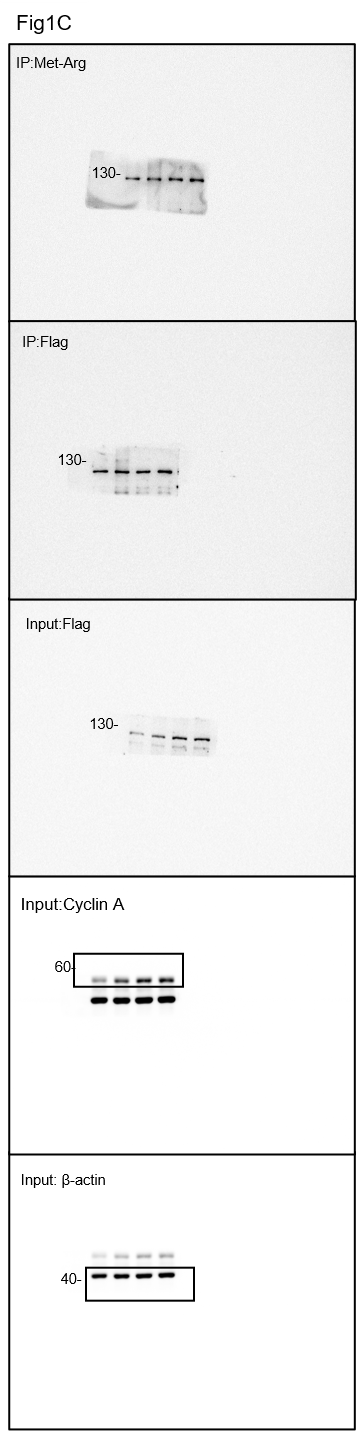

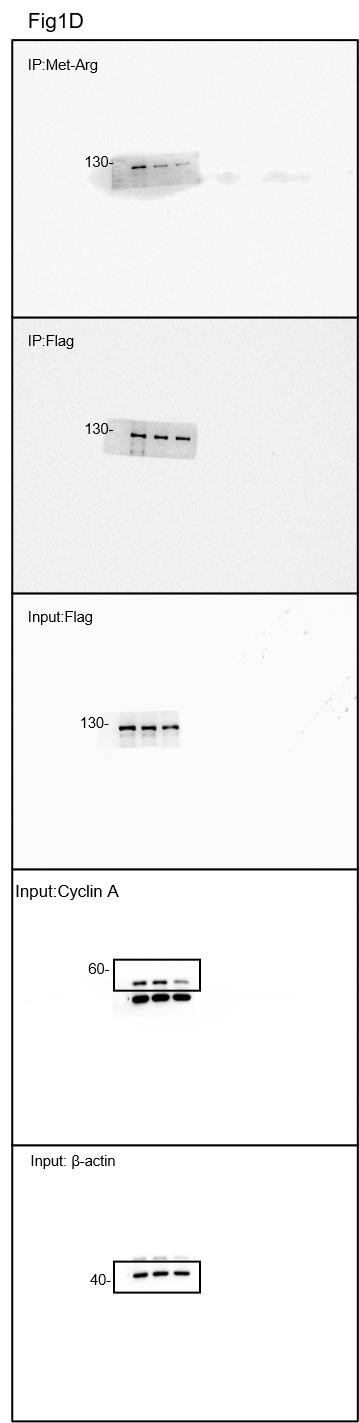

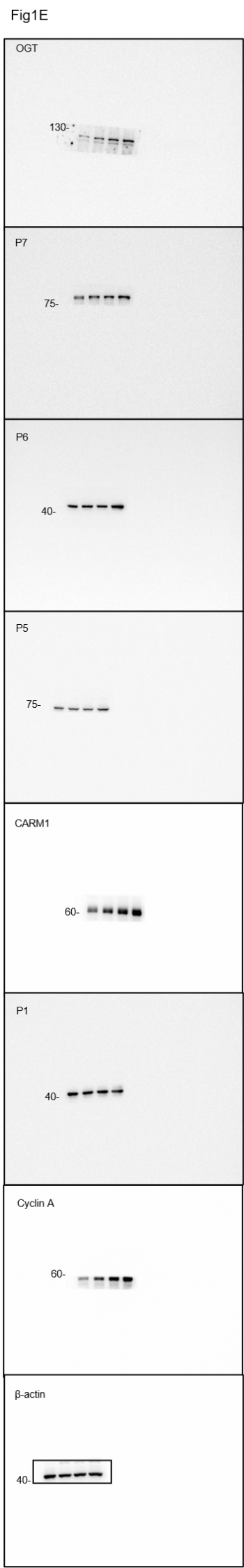

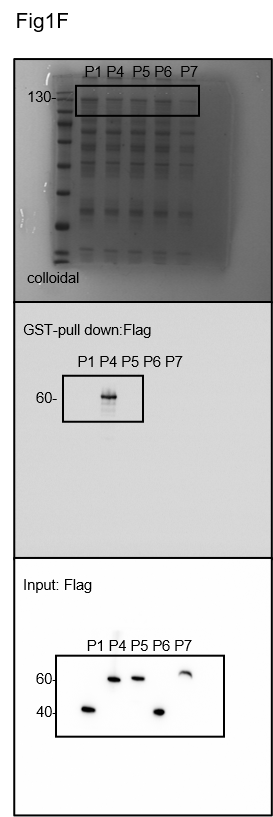

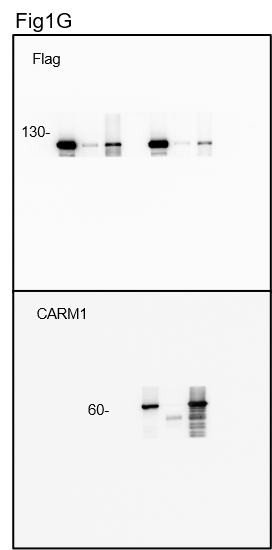

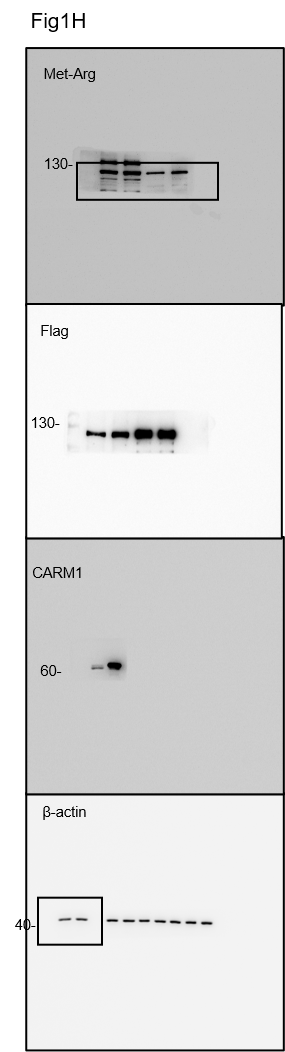

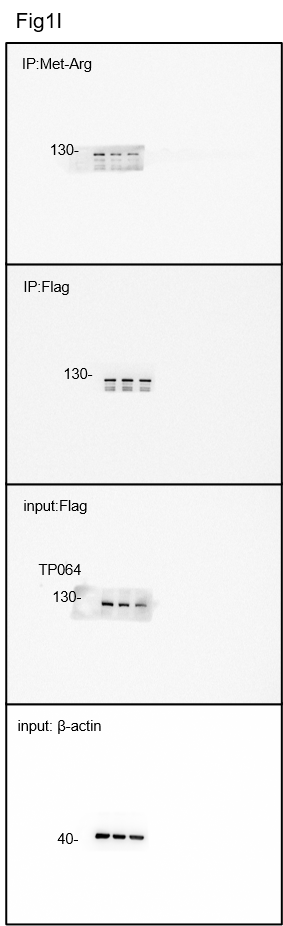

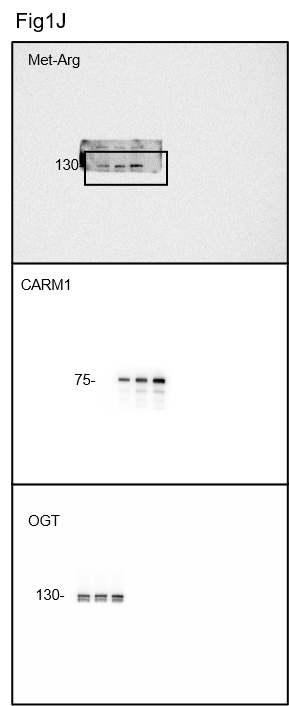

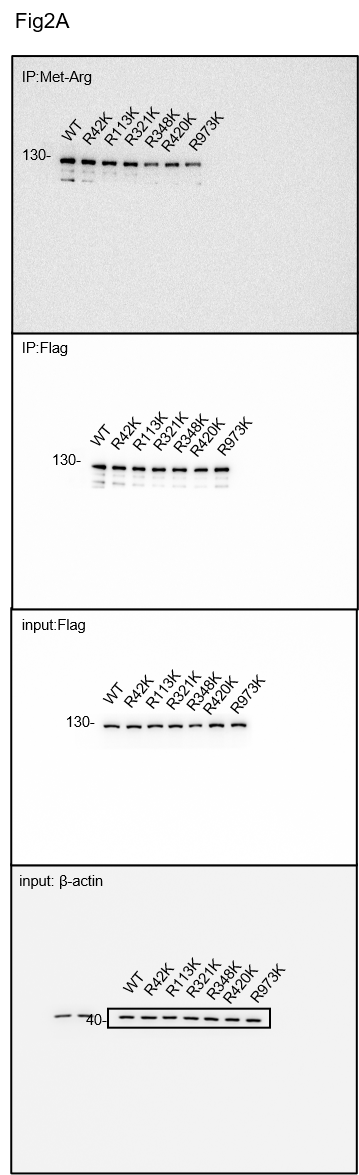

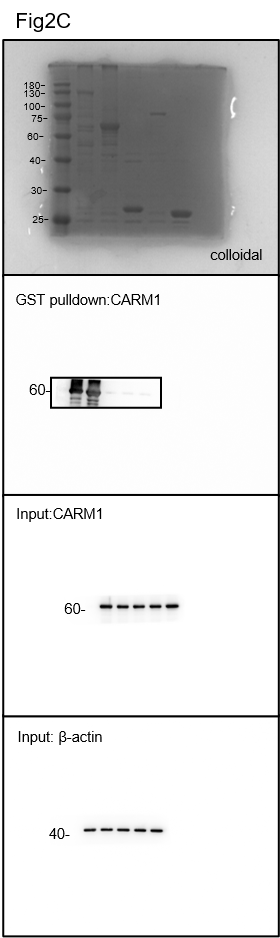

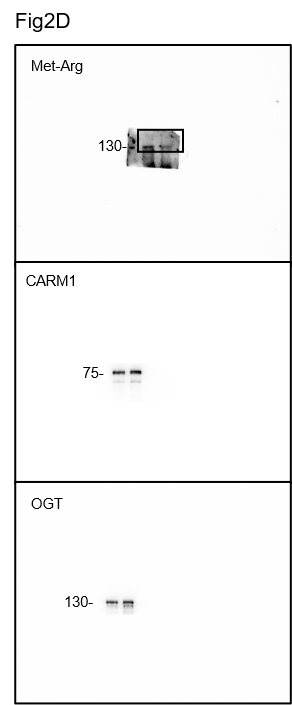

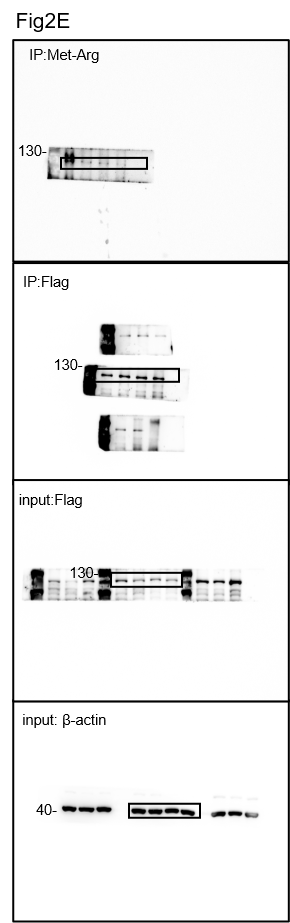

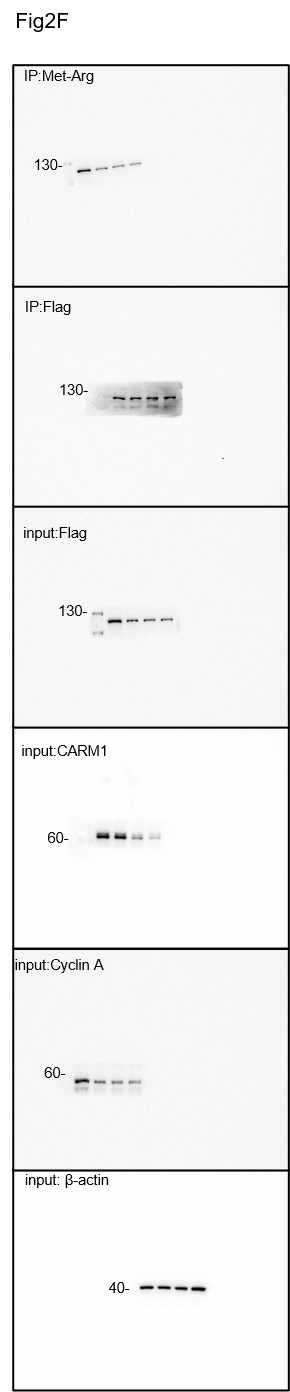


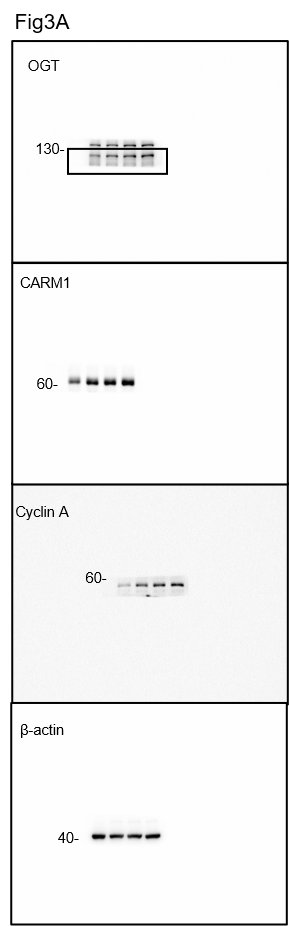

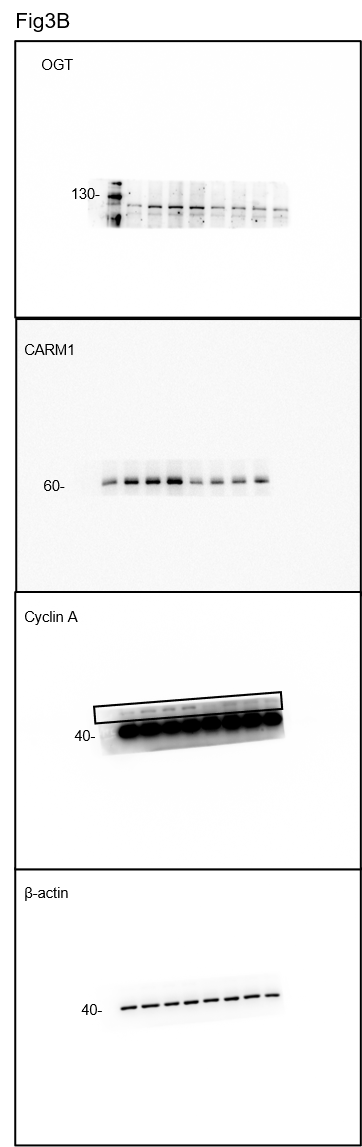

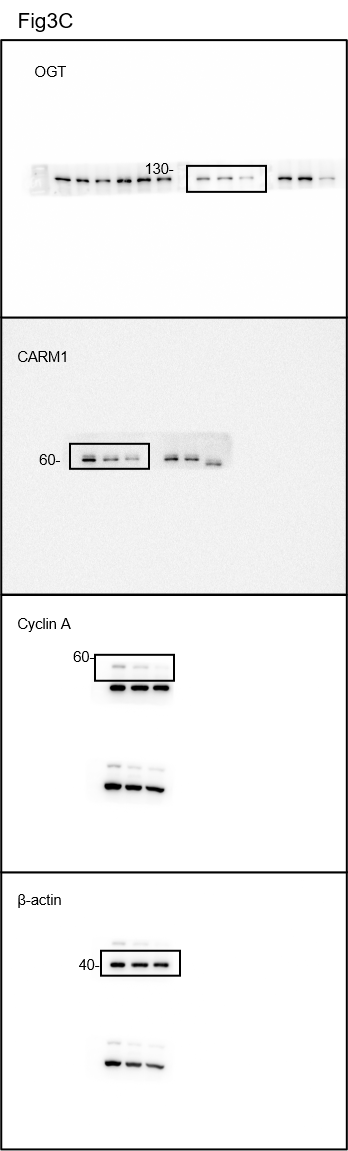

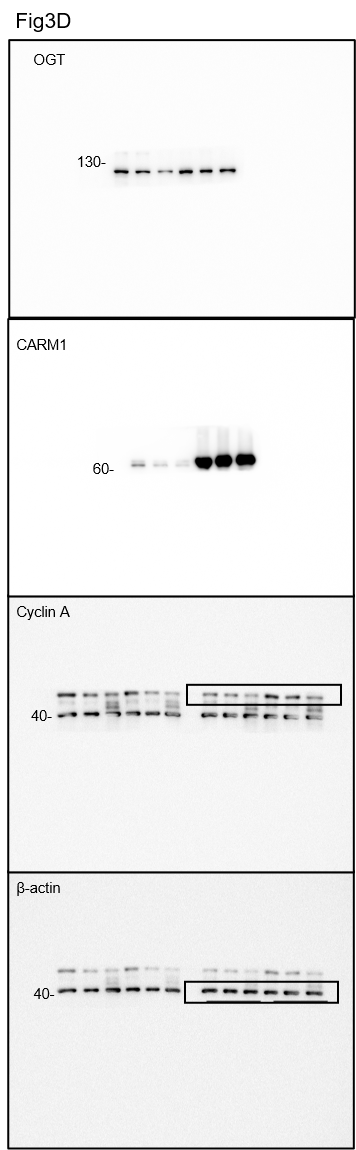

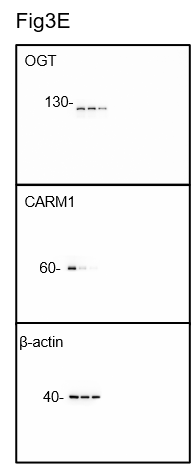

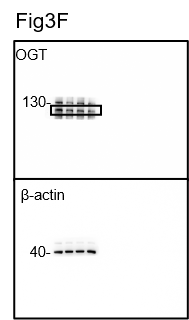

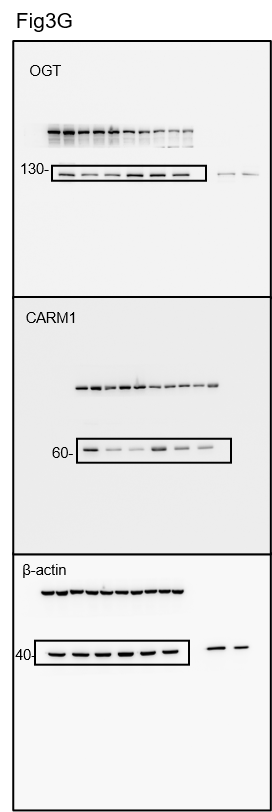

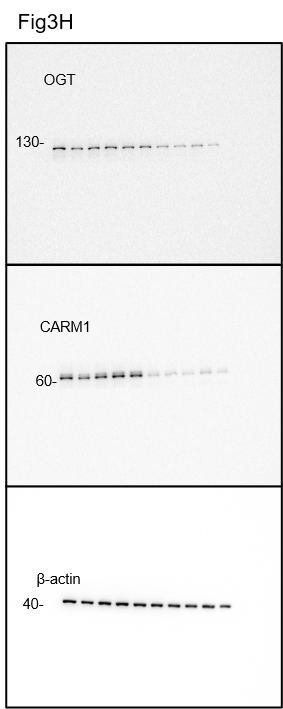

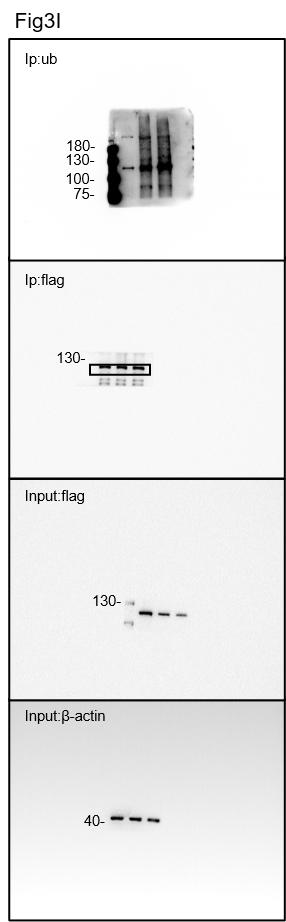

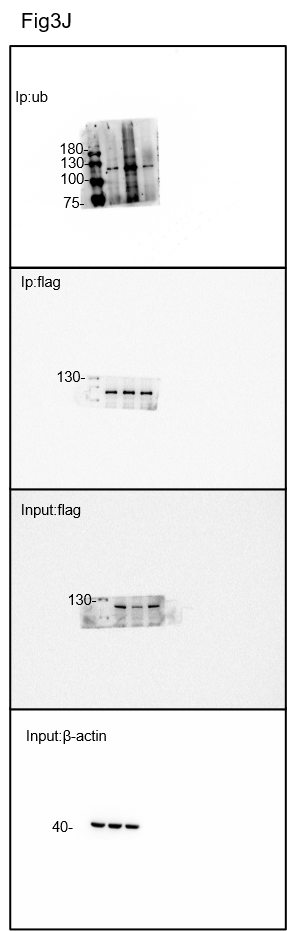

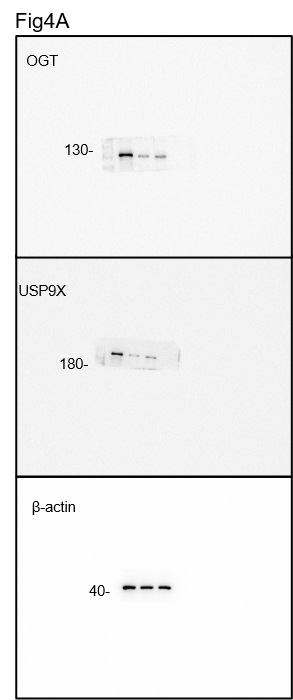

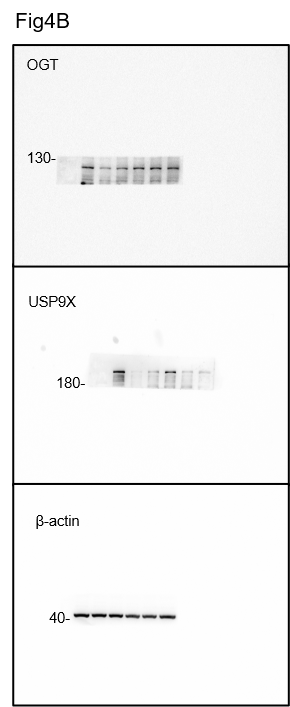

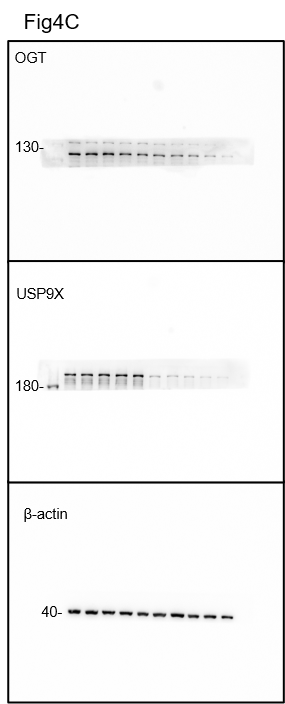

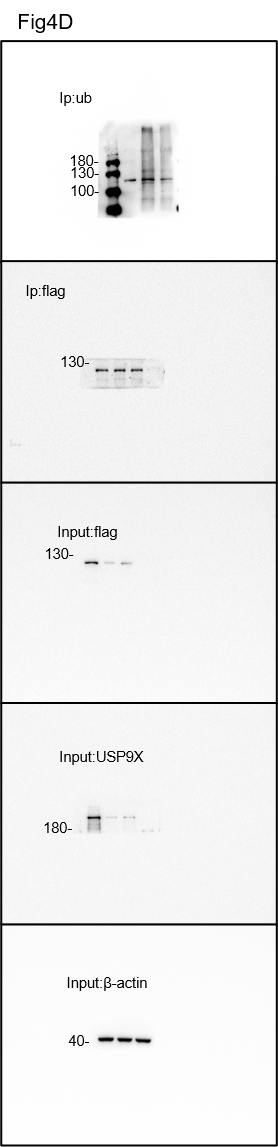

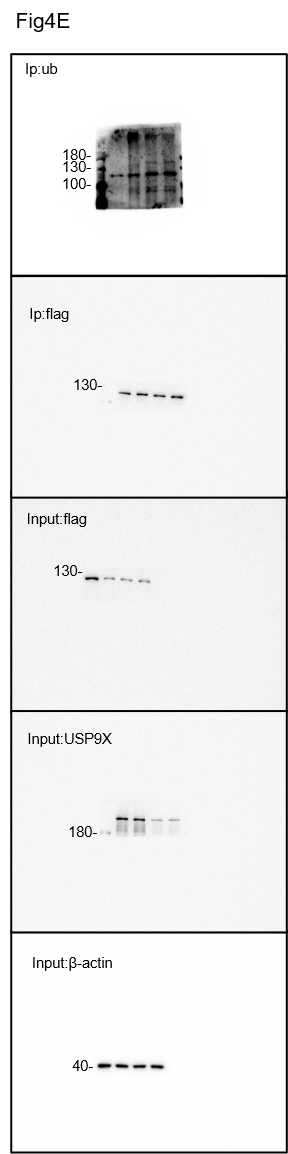

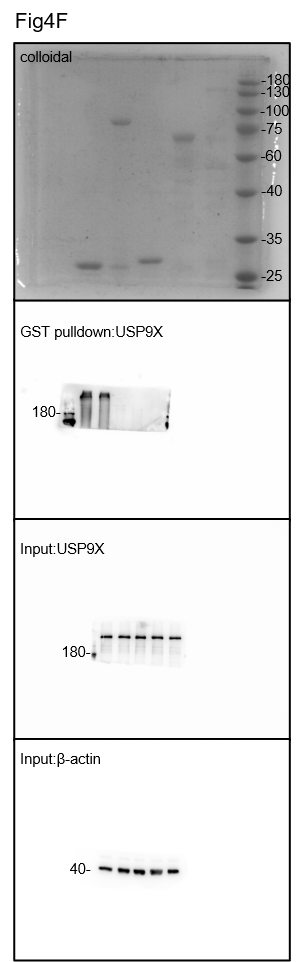

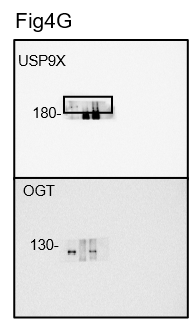

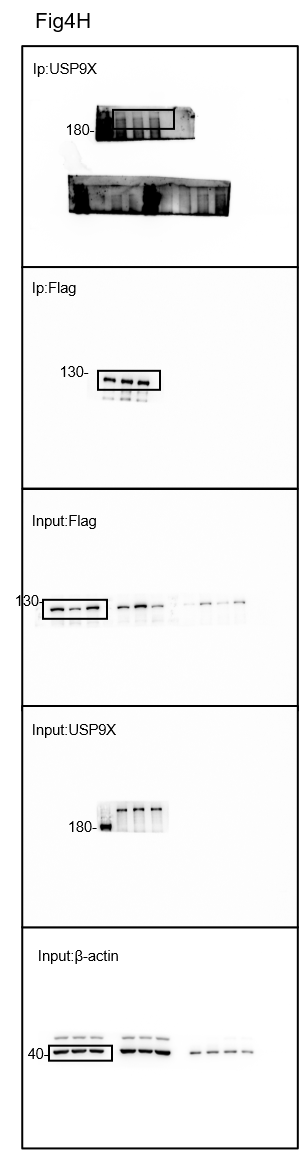

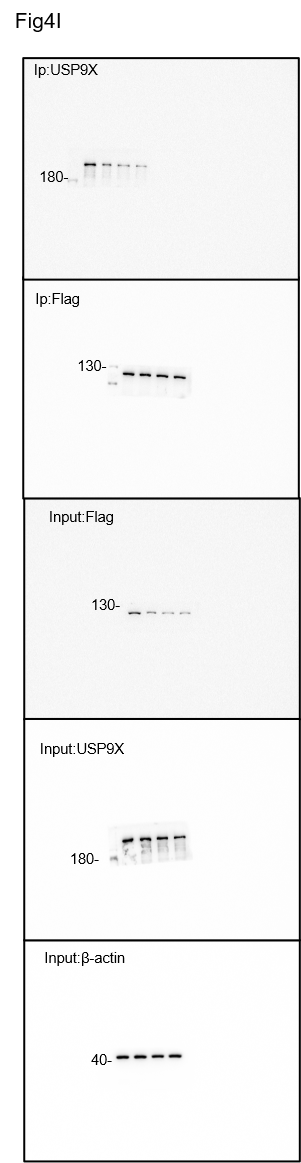


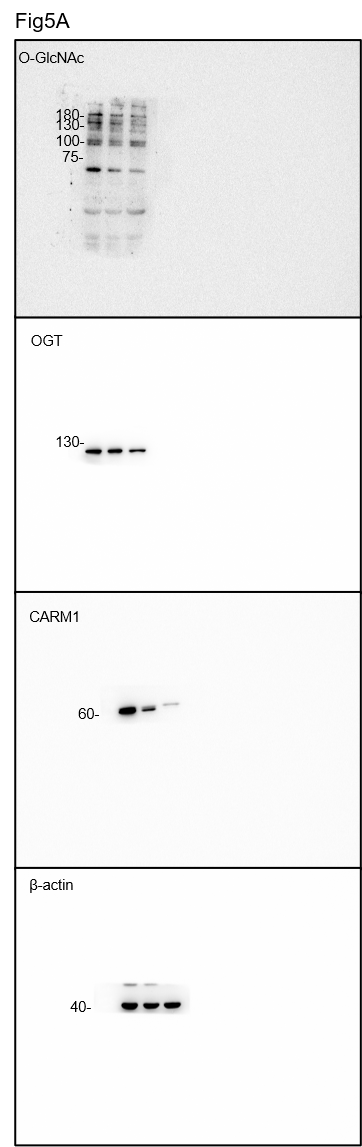

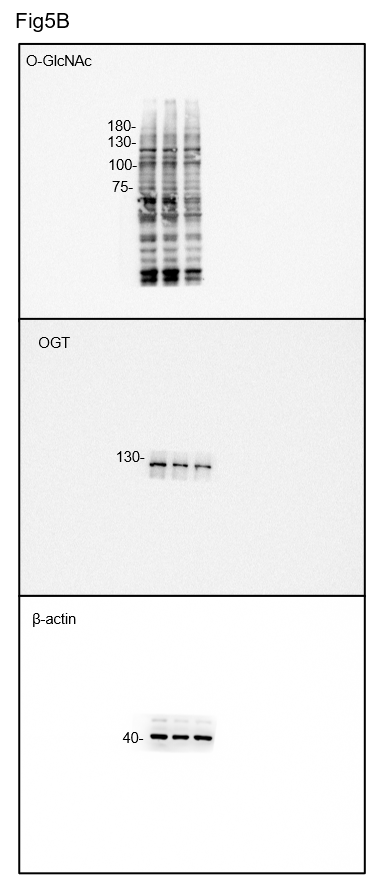

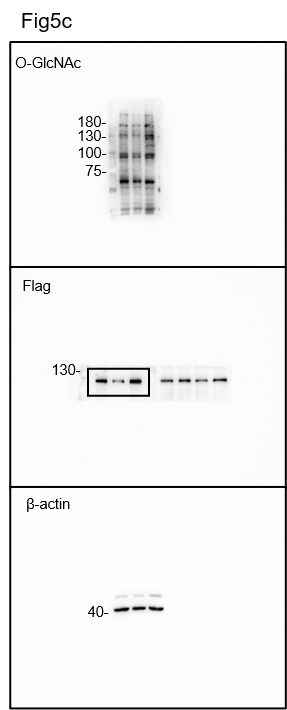

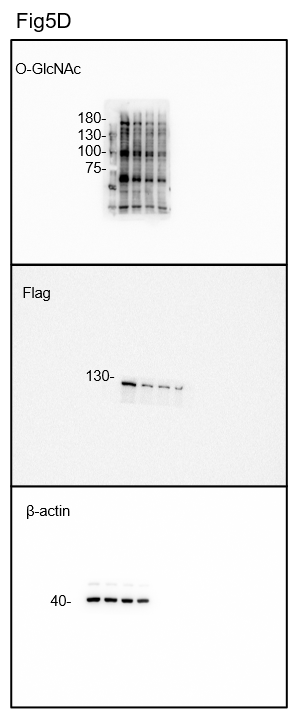


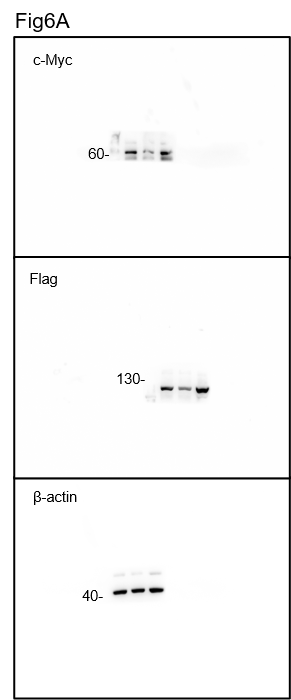

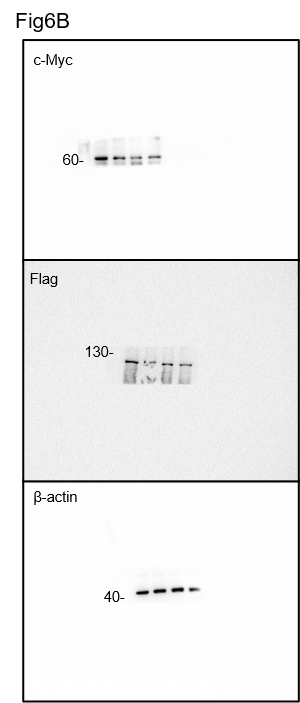


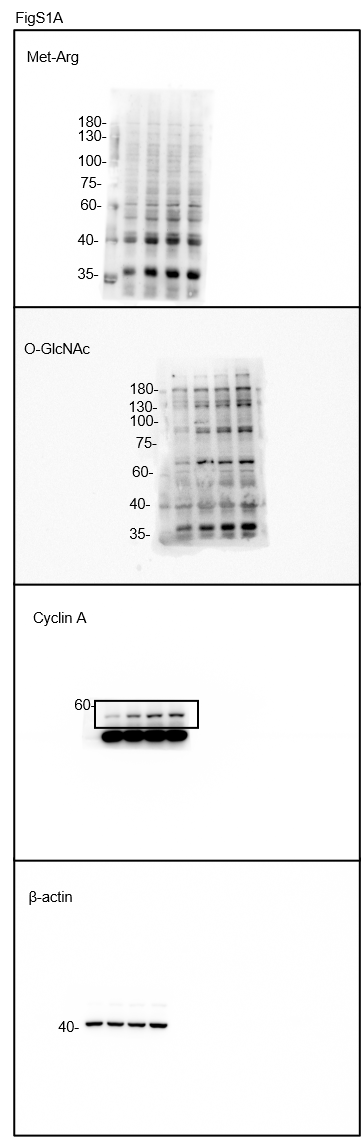


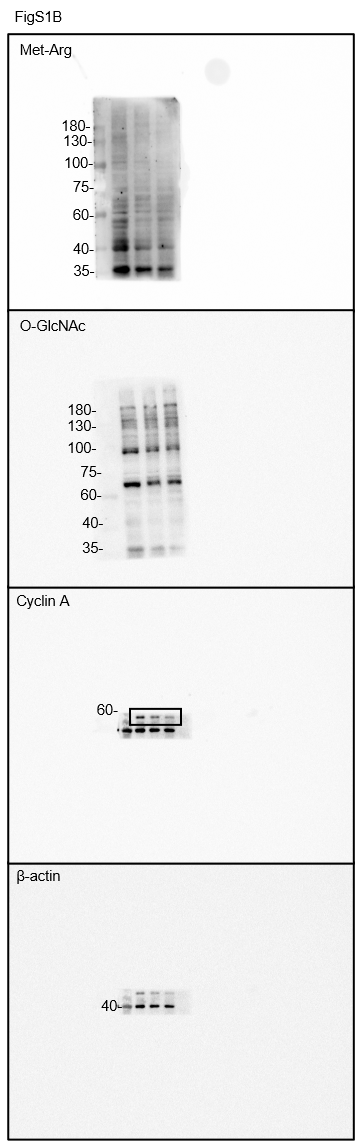

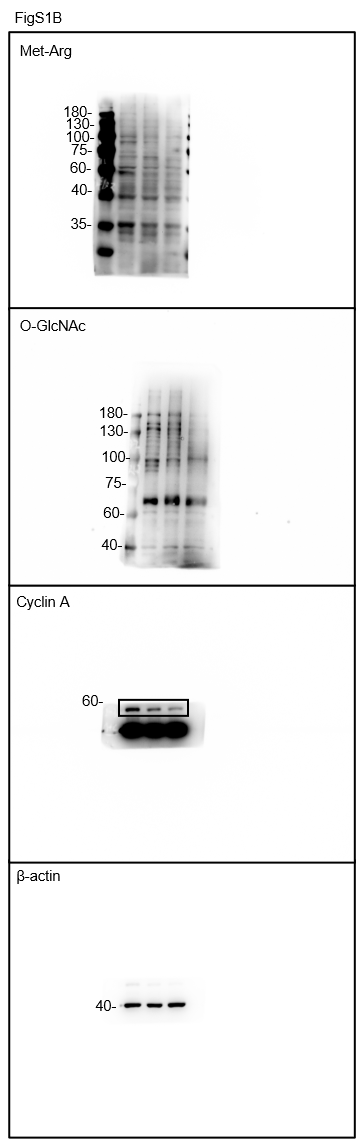


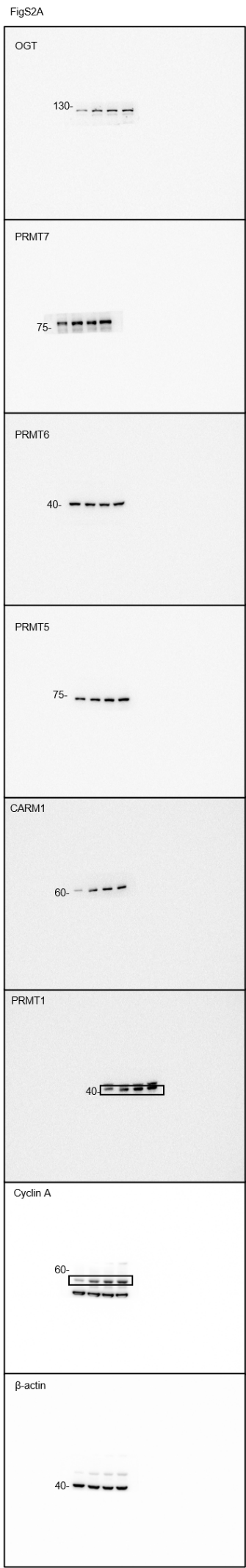

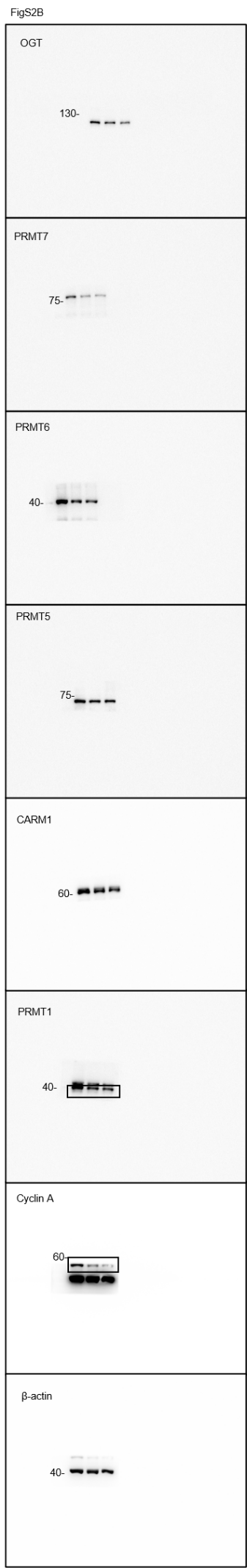

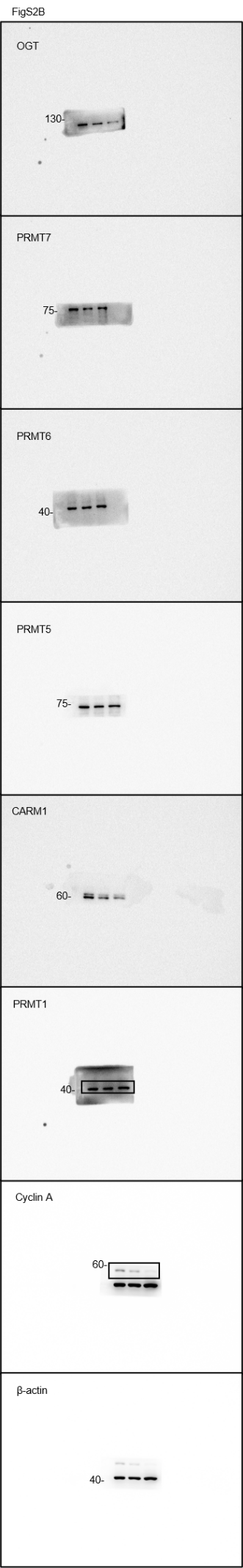


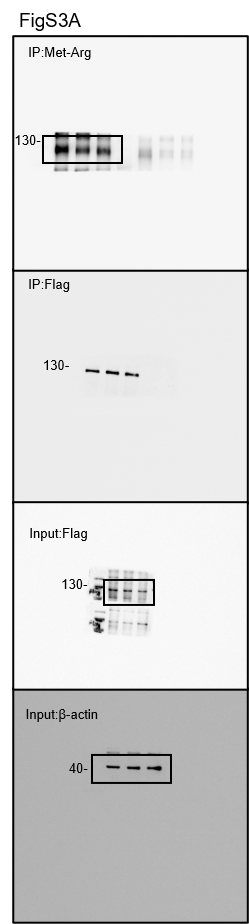


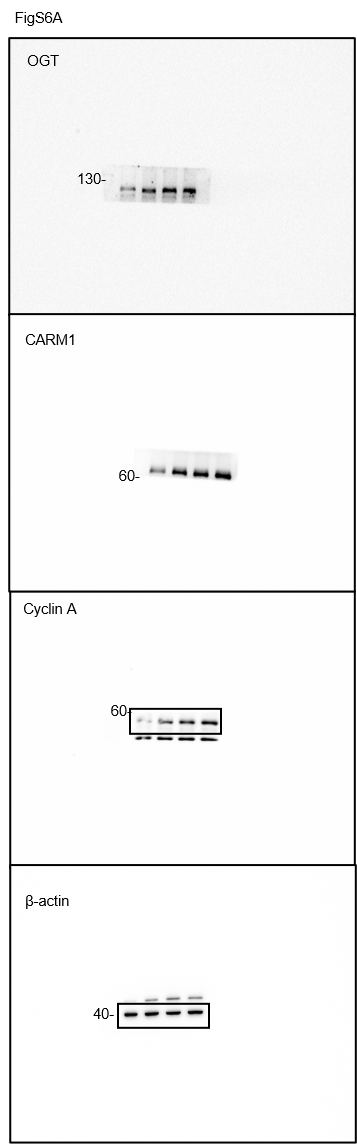

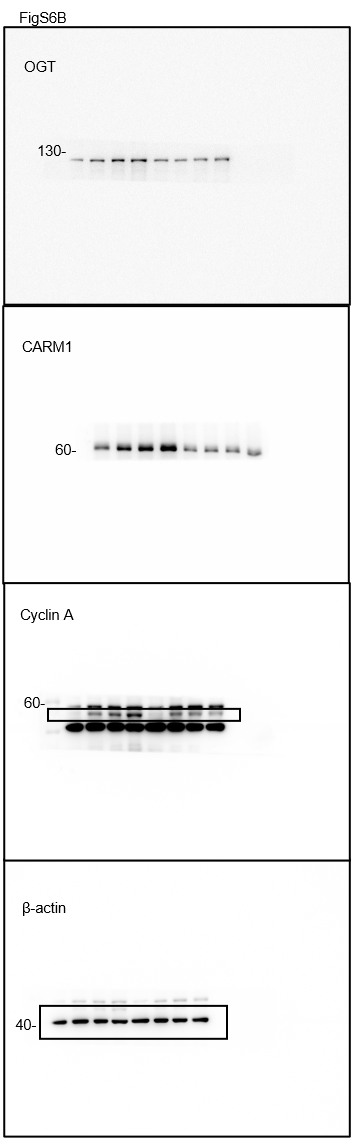

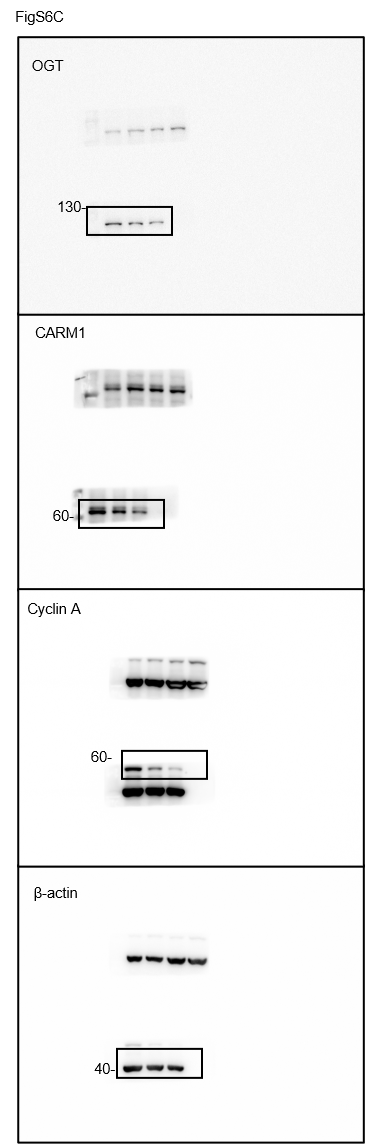

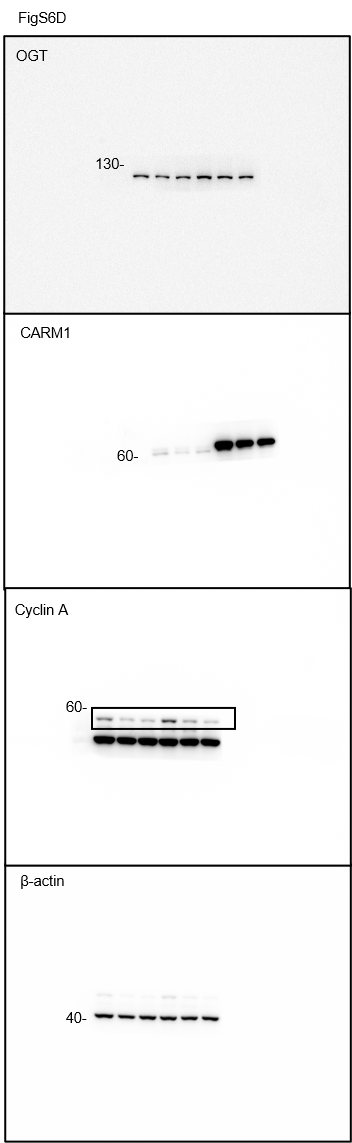

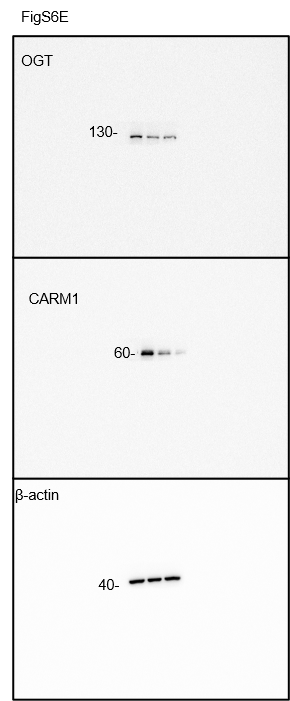

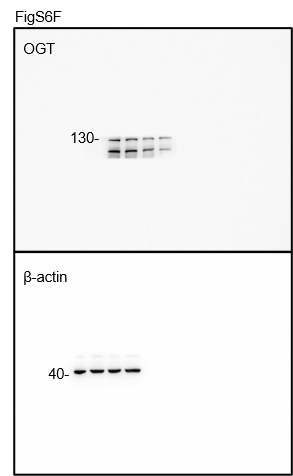

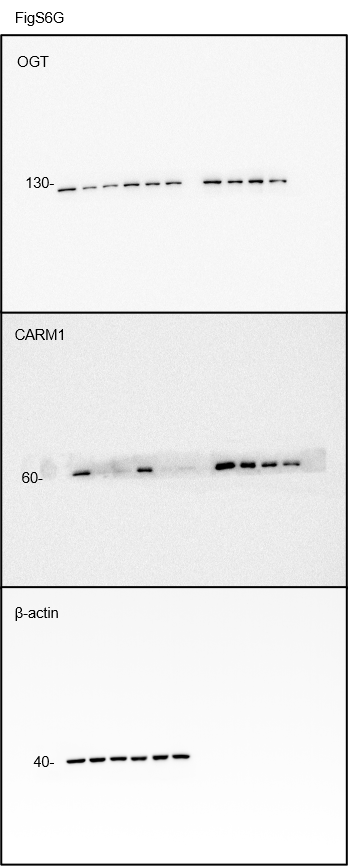

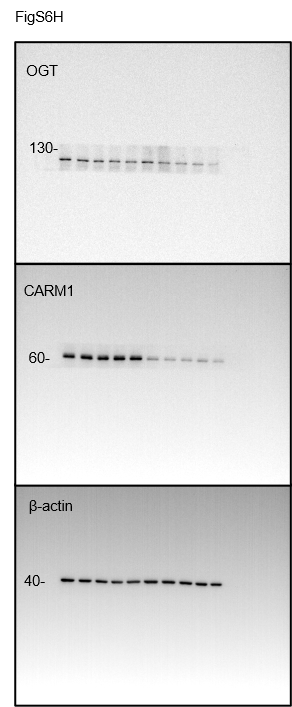


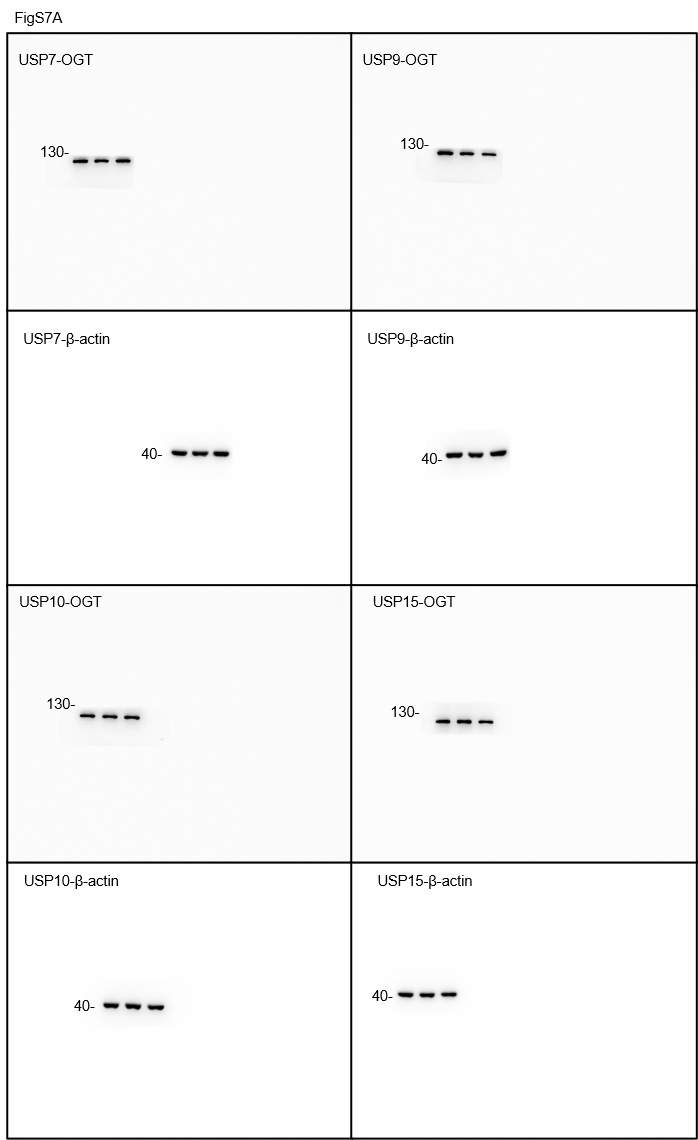

Supplement: Supplementary file 12 — WB blot [file 41419_2024_7313_MOESM12_ESM.docx]
