# Supplementary material for: CARM1-mediated OGT arginine methylation promotes non-small cell lung cancer glycolysis by stabilizing OGT
Source: Cell Death Dis. 2024 Dec 23;15(12):927. doi: 10.1038/s41419-024-07313-1 (PMC11666572; doi:10.1038/s41419-024-07313-1)
Supplement: Supplementary file 9 — Supplementary Materials and Methods [file 41419_2024_7313_MOESM9_ESM.docx]

Supplementary Materials and Methods

**Antibodies, reagents and plasmids**

All the reagents used were purchased from Sangon Biotech(Shanghai) except that listed below (Reagent, Manufacturer Catalogue Number).

ASYM, Millipore 07-414; Mer-Arg, Abcam ab412; O-GlcNAc, Abcam ab2739; Cyclin A, Abclonal A7632; β-actin, Abclonal AC026; Flag, Abmart M20008; OGT (IHC), Abclonal A2214; OGT, Abclonal A3501; OGT (Co-IP), CST 24083; PRMT1, Abclonal A4502; CARM1, Millipore 09-818; PRMT5, Millipore 07-405; PRMT6, CST 14641; PRMT7, CST 14762S; Ubquitin, CST 3933; USP9X, Proteintech 55054-1-AP; c-Myc, Abclonal A1309; normal rabbit IgG, CST 2729; OGA, Abclonal A24124; normal mouse IgG,Santa Cruz Biotechnology SC-2025; H3R17me2a, Abcam ab8284.

CCK8, APEBIO K1018; ADOX, Selleck S8608; Glucose, Sigma G7528; TP064, TOCRIS 6008; SAM, Sigma A2408; DMSO, Sigma DH105; MG132, Beyotime S1748; CHX, Sigma C104450; DMEM, Gibico 11966025.

His-tagged OGT plasmid was used to generate Flag-tagged OGT in the pCDH-CMV-3×Flag vector. Flag-tagged OGT was used to generate Flag-tagged OGT R42K/R113K/R321K/R348K/R420K/R973K/R348F. All mutations were verified by DNA sequencing in Comate Bioscience (Changchun, China).

Bacterial expression plasmids expressing GST-tagged OGT (pGEX-6P-1-OGT) were generated by inserting the target DNA fragments into the pGEX-6P-1 vector in frame with the GST coding sequence.

Other vectors used in this study were as follows: pWPXLd-CARM1, pWPXLd-Flag-PRMT1/CARM1/PRMT5/PRMT6/PRMT7, and pGEX-6P-1-CARM1.

All the restriction enzymes used were product of New England BioLabs (NEB. Inc.) and were as follows: BamH1-HF (R3136); Not I-HF (R3189); EcoRI-HF (R3101); AgeI-HF (R3552). T4 DNA Ligase was also an NEB product (M0202).

The shRNA sequences were found on Sigma-Aldrich official website and tested in the NCBI BLAST system to ensure specificity.The sequences used are listed below (5’-3’ , sense, antisense).

shCARM1#1,CCGGGGACATGTCTGCTTATTGCCTCGAGGCAATAAGCAGACATGTCCTTTTTG,AATTCAAAAAGGACATGTCTGCTTATTGCCTCGAGGCAATAAGCAGACATGTCC;

shCARM1#2,CCGGCTATGACTTGAGCAGTGTTATCTCGAGATAACACTGCTCAAGTCATAGTTTTTG,AATTCAAAAACTATGACTTGAGCAGTGTTATCTCGAGATAACACTGCTCAAGTCATAG;

shUSP7#1,CCGGCCTGGATTTGTGGTTACGTTACTCGAGTAACGTAACCACAAATCCAGGTTTTTG,AATTCAAAAACCTGGATTTGTGGTTACGTTACTCGAGTAACGTAACCACAAATCCAGG;

shUSP7#3,CCGGCGTGGTGTCAAGGTGTACTAACTCGAGTTAGTACACCTTGACACCACGTTTTTG,AATTCAAAAACGTGGTGTCAAGGTGTACTAACTCGAGTTAGTACACCTTGACACCACG;

shUSP9#1,CCGGGAGAGTTTATTCACTGTCTTACTCGAGTAAGACAGTGAATAAACTCTCTTTTTG,AATTCAAAAAGAGAGTTTATTCACTGTCTTACTCGAGTAAGACAGTGAATAAACTCTC;

shUSP9#2,CCGGCGCCTGATTCTTCCAATGAAACTCGAGTTTCATTGGAAGAATCAGGCGTTTTTG,AATTCAAAAACGCCTGATTCTTCCAATGAAACTCGAGTTTCATTGGAAGAATCAGGCG;

shUSP10#1,CCGGCCTATGTGGAAACTAAGTATTCTCGAGAATACTTAGTTTCCACATAGGTTTTTG,AATTCAAAAACCTATGTGGAAACTAAGTATTCTCGAGAATACTTAGTTTCCACATAGG;

shUSP10#2,CCGGCCCATGATAGACAGCTTTGTTCTCGAGAACAAAGCTGTCTATCATGGGTTTTTG,AATTCAAAAACCCATGATAGACAGCTTTGTTCTCGAGAACAAAGCTGTCTATCATGGG;

shUSP15#1,CCGGGCTCTTGAGAATGTGCCGATACTCGAGTATCGGCACATTCTCAAGAGCTTTTTG,AATTCAAAAAGCTCTTGAGAATGTGCCGATACTCGAGTATCGGCACATTCTCAAGAGC;

shUSP15#2,CCGGCCTTGGAAGTTTACTTAGTTACTCGAGTAACTAAGTAAACTTCCAAGGTTTTTG,AATTCAAAAACCTTGGAAGTTTACTTAGTTACTCGAGTAACTAAGTAAACTTCCAAGG;

shOGT-3’UTR-5, CCGGGCACATCATTCCTCCTATAGGCTCGAGCCTATAGGAGGAATGATGTGCTTTTTG,AATTCAAAAAGCACATCATTCCTCCTATAGGCTCGAGCCTATAGGAGGAATGATGTGC;

shOGT 3’UTR-6, CCGGGCCAGCATGGTTGCAGATAAACTCGAGTTTATCTGCAACCATGCTGGCTTTTTG,AATTCAAAAAGCCAGCATGGTTGCAGATAAACTCGAGTTTATCTGCAACCATGCTGGC.

**Reverse transcription, PCR, and real-time PCR**

The sequences of PCR primers are listed as follows (5’-3’, sense, antisense): β-actin, GAGCACAGAGCCTCGCCTTT; ATCCTTCTGACCCATGCCCA; CARM1, TCGCCACACCCAACGATTT; GTACTGCACGGCAGAAGACT; USP9X, TCGGAGGGAATGACAACCAG; GGAGTTGCCGGGGAATTTTCA; USP7, GGAAGCGGGAGATACAGATGA; AAGGACCGACTCACTCAGTCT; USP10, ATGATTCTAAGCCCTCTGCCTCCT; ATTCATGAGCCGAACAAAGCTATC; USP15, TGCCTACTTCCAACTCTC; GCTCTTCCTTTCCTTCTC; OGT, TGCTTGGACACTCCACTCTG; GAGCCGCTCTAGTTCCATTG.

**Measurement of extracellular acidification rate (ECAR) and lactate**

ECAR was measured in an XF96 Extracellular Flux Analyzer (Seahorse Bioscience, North Billerica, MA, USA). Cells were seeded into XFp Cell Culture Miniplates and incubated in complete medium. The next day, the medium was changed to analysis medium and incubated in a CO_2_- free incubator at 37°C for 1 h. Cells were sequentially exposed to 100 mM glucose, 10 μM oligomycin, and 500 mM 2-Deoxy-D-glucose (2-DG).

Lactate concentration was measured using the Lactic Acid (LA) Content Assay Kit (Solarbio, China) according to the manufacturer’s instructions.

**CCK8 and clone formation assays**

For the CCK8 assay, cells were seeded into 96 well microplates at a density of 1 × 10^3^. Cell viability was assessed by the CCK8 assay at the indicated time points and absorbance was measured at 450 nm.

For the clone formation assay, cells (1 × 10^3^) were seeded into 3 cm culture plates. After 10 days, the clones were stained with 0.1% crystal violet, photographed and counted.

**supplementary figure legend**

Fig S1. O-GlcNAcylation and arginine methylation levels change consistently in correlation with changes in glucose in NSCLC.

A. A549 cells were cultured for 18 h with different concentrations of glucose as indicated. O-GlcNAcylation and arginine methylation levels were analyzed by western blotting. B. A549 cells and H1299 cells were cultured with glucose starvation for the indicated times. O-GlcNAcylation and arginine methylation levels were analyzed by western blotting.

Fig S2. CARM1 level changes consistently in correlation with changes in glucose in NSCLC.

A. A549 cells were cultured with different concentrations of glucose for 18 h. PRMT and OGT levels were analyzed by western blotting. B. A549 cells and H1299 cells were cultured with glucose starvation for different times. OGT and CARM1 levels were analyzed by western blotting.

Fig S3. OGT is arginine methylated.

A. HEK-293T cells overexpressing Flag-tagged OGT were treated with increasing concentrations of ADOX for 24 h as indicated. Co-immunoprecipitation (Co-IP) was then performed using anti-Flag antibody. Arginine methylation of immunopurified OGT was detected with antibodies as indicated.

Fig S4. Six methylated arginine residues (Arg 42/113/321/348/420/973) are identified.

A. Mass spectrometric analysis of OGT methylation. Co-IP was performed using anti‐Flag antibody in HEK‐293T cells overexpressing Flag‐tagged OGT, followed by liquid chromatography coupled with tandem mass spectrometry (LC‐MS/MS) analysis.

Fig S5. OGT R348 and OGT R973 are located at a considerable distance from each other.

A. Ribbon diagram of OGT showing its structure with R348 and R973 residues.

Fig S6. CARM1 modulates the stability of OGT through the proteasome pathway in A549 cells.

A. A549 cells were cultured with different concentrations of glucose for 18 h. CARM1 and OGT levels were analyzed by western blotting. B. A549 cells were transfected with control or CARM1 shRNA#2 and then cultured with different concentrations of glucose for 18 h. CARM1 and OGT levels were analyzed by western blotting. C. A549 cells were cultured with glucose starvation for the indicated times. OGT and CARM1 levels were analyzed by western blotting. D. A549 cells were transfected with vector or CARM1 and then cultured with the glucose starvation for different times. CARM1 and OGT levels were analyzed by western blotting. E. The protein expression levels of OGT in A549 cells transfected with CARM1 shRNAs or control vector were assessed by western blotting. F. A549 cells were treated with the indicated amounts of TP064 for 24 h. The protein expression level of OGT in cells were assessed by western blotting. G. A549 cells were transfected with control or CARM1 shRNAs and then treated with MG132 or DMSO for 8 h, and the cell lysates were analyzed by western blotting. H. A549 cells were transfected with CARM1 shRNA#2 or control vector, and then treated with CHX (100 μg/mL) for the indicated time; OGT protein level was examined by western blotting.

Fig S7. OGT downregulation is most pronounced after knockdown of USP9X.

A. The protein expression levels of OGT in HEK-293T cells transfected with USPs shRNAs or control were assessed by western blotting. Relative mRNA expression levels of USPs in HEK-293T cells transfected with USPs shRNAs or control were assessed by qPCR (error bars represent the mean ± SD, n = 3 experimental replicates, *P < 0.05, **P < 0.01, ***P < 0.001, ****P < 0.0001, Student's t-test).

Fig S8. The regulation of CARM1 by glucose conditions is carried out at the post-transcriptional level.

A. H1299 cells were cultured for 18 h with different concentrations of glucose as indicated. Relative mRNA expression levels of CARM1 was assessed by qPCR (error bars represent the mean ± SD, n = 3 experimental replicates, ns = not significant, Student's t-test). B. H1299 cells were cultured with glucose starvation for the indicated times. Relative mRNA expression levels of CARM1 was assessed by qPCR (error bars represent the mean ± SD, n = 3 experimental replicates, ns = not significant, Student's t-test).
